# Supplementary material for: Achieving health-oriented air pollution control requires integrating unequal toxicities of industrial particles
Source: Nat Commun. 2023 Oct 14;14:6491. doi: 10.1038/s41467-023-42089-6 (PMC10576764; doi:10.1038/s41467-023-42089-6)
Supplement: Supplementary file 1 — Supplementary Information [file 41467_2023_42089_MOESM1_ESM.pdf]

Supplementary Materials for

Achieving health-oriented air pollution control requires integrating unequal toxicities of industrial particles

Di Wu,[1,#] Haotian Zheng,[2,3,#] Qing Li,[1,4,*] Shuxiao Wang,[2,3,*] Bin Zhao,[2,3] Ling Jin,[5,6] Rui Lyu,[7] Shengyue Li,[2] Yuzhe Liu,[1] Xiu Chen,[1] Fenfen Zhang,[2] Qingru Wu,[2,3] Tonghao Liu,[8] Jingkun Jiang,[2,3] Lin Wang,[1] Xiangdong Li,[5] Jianmin Chen,[1,4] and Jiming Hao[2,3]

[1]Department of Environmental Science and Engineering, Shanghai Key Laboratory of Atmospheric Particle Pollution and Prevention, Fudan University, Shanghai 200433, China

[2]State Key Joint Laboratory of Environment Simulation and Pollution Control, School of Environment, Tsinghua University, Beijing 100084, China

[3]State Environmental Protection Key Laboratory of Sources and Control of Air Pollution Complex, Beijing 100084, China

[4]Shanghai Institute of Eco-Chongming (SIEC), 20 Cuiniao Road, Chenjia Town, Chongming District, Shanghai 202162, China.

[5]Department of Civil and Environmental Engineering, The Hong Kong Polytechnic University, Hong Kong, China

[6]Department of Health Technology and Informatics, The Hong Kong Polytechnic University, Hong Kong, China

[7]China Huaneng Clean Energy Research Institute, Beijing, 102209, China

[8]China National Environmental Monitoring Center, Beijing, 100012, China

#These authors contributed equally.

[*] Correspondence: qli@fudan.edu.cn or shxwang@tsinghua.edu.cn

The Supplementary Materials include the following sections:

Supplementary Notes: 1 to 11

Supplementary Figures: 1 to 15

Supplementary Tables: 1 to 18

Supplementary References

Supplementary Notes

1. Nationwide Control Measures for Industrial Sectors

Iron and steel industry (ISI), power industry, and cement industry were chosen as the target industries for this study due to their significant contributions to ambient $PM_{2.5}$ pollution worldwide. These three sectors were the top polluting sectors among all industry sources, accounting for 15.1% of global total anthropogenic $PM_{2.5}$ emissions[1]. The source apportionment of ambient $PM_{2.5}$ in China from 2005 to 2015 also suggested that power industry and industrial processes (mainly consist of ISI and cement industry) contributed 34%, 32%, and 24% in 2005, 2010, and 2015, respectively[2, 3]. Their contributions could be even higher in key regions, such as Beijing-Tianjin-Hebei region, Yangtze River Delta, and Pearl River Delta. Therefore, analyzing the environmental impact of these sectors is essential to better design emission control policies in China, particularly from the perspective of toxic potency.

To reduce air pollution originating from industrial sources, China has enforced a series of control measures targeting industrial sectors over the past few decades (Supplementary Table 8). These control policies initially focused on power plants (GB13223-91[4] issued in 1991, GB13223-1996[5] issued in 1996, and GB13223-2003[6] issued in 2003) and were then extended to other industrial sectors, especially after 2010. China implemented the GB 13223-2011[7] emission standard in 2012 to limit particulate matter (PM) emission concentrations stemming from coal-fired power plants (CFPPs) to 30 mg/m^3 at the 6% standard oxygen level, which was fully met by all CFPPs at the end of 2015[8, 9]. To further reduce PM emissions, China enforced the toughest-ever ultralow emission (ULE) standards for CFPPs in 2014, which further limited the PM emission concentration to 10 mg/m^3. Existing dedusting devices have been continuously upgraded into high-efficiency devices to meet ULE requirements (Supplementary Table 9)[10]. The PM emission levels required by the ULE standards in mainland China are already lower than those required in the United States (22.5 μg/m^3 for PM) and close to those required in the European Union (2−18 μg/m^3) (Supplementary Table 9). By the end of 2017, all CFPP units had already satisfied the emission standards (GB 13223-2011)[7], while 72.3% of all CFPPs in mainland China had met the ULE standards[11].

China has progressively promulgated several editions of air pollution control standards (GB 4911-1985[12] issued in 1985, GB 9078-1996[13] issued in 1996, GB 28662-2012[14] issued in 2012, and GB 28663-2012[15] issued in 2012, GB 28664-2012[16] issued in 2012, GB 20665-2012[17] issued in

2012) targeting the iron and steel industry (ISI) over the past few decades. To promote air pollution reduction, China imposed ever-tightening emission standard policies for the major iron and steel production processes in 2012, including sintering and pelletizing (GB 28662-2012)[14], ironmaking (GB 28663-2012)[15], steelmaking (GB 28664-2012)[16], and steel rolling (GB 20665-2012)[17]. The upper limits of PM emissions from major stationary sources ranges from 15−50 mg/m^3 for new plants and from 20−100 mg/m^3 for existing plants. These emission standards were then strengthened in January 2015, lowering the upper limits of PM emissions by as much as 60%. Further detailed information can be found in a previous study[18]. China announced opinions on advancing the implementation of ULE standards in the ISI in April 2019, which limits the PM concentration to 10 mg/m^3 among organized emission sources[19]. Opinions have been prioritized on iron and steel plants located in key regions (i.e., Beijing-Tianjin-Hebei and surrounding areas, the Yangtze River Delta region, and the Fen-Wei Plain). The PM emission levels required by the 2012 standard are higher than those required in the European Union and United States (Supplementary Table 10). However, the PM emission limits contained in the ULE standards are at the same level as those currently implemented in the European Union and United States and even exceed the PM emission standards for sintering processes.

Four editions of PM emission standards (GB 4915-1985[20], GB 4915-1996[21], GB 4915-2004[22], and GB 4915-2013[23]) were progressively enforced in the cement industry since 1985 in mainland China[24]. Compared to the PM emission standard announced in 2004 (GB 4915-2004)[22] for the cement industry, the upper limits of PM emissions were reduced by as much as 40% in the stricter standard announced in December 2013 (GB 4915-2013)[23] and enforced since July 2015[23]. The emission standards (GB 4915-2013)[23] limit the PM concentration to 10−20 mg/m^3 in key regions and 20−30 mg/m^3 in other regions (Supplementary Table 11). The limit levels of the current PM emission standards for the cement industry are higher than those of prevailing standards in the European Union (10−20 mg/m^3) and United States (4−14 mg/m^3). Furthermore, more stringent local PM emission standards, namely, the ULE standards, were progressively introduced in six key provinces (namely, Hebei, Anhui, Henan, Hainan, Jiangsu, and Sichuan) in 2020[25], which limit the PM concentration to 10 mg/Nm^3. The PM limits of the ULE standards in local provinces are lower than those listed in the current European Union standards.

In China, industrial emission control standards were initially employed targeting CFPPs and were then extended to other industrial sources based on total PM mass emission control strategies,

which are similar to those practiced in developed countries. Some developing regions still experienced severe industrial PM pollution, and they have begun to promote mass-based emission control standards for air quality improvement purposes, such as India and Brazil. The end-of-pipe PM emission control standards for the three industrial sources were progressively strengthened due to the ever-tightening ambient $PM_{2.5}$ standards. The toughest ULE standards were first implemented in the nationwide power sector among the industrial sectors, followed by the ISI.

2. Studied Iron and Steel Units

The ISI exhibited a total of 1885 plant processes operating nationwide in 2018 in mainland China[18]. Most of the iron and steel plants are located in densely populated areas (such as Hebei, Shandong, and Jiangsu provinces). Two routes for crude steel production are commonly employed in China, including (1) producing pig iron in a blast furnace (BF), which is followed by steel production in a basic oxygen furnace (BOF), and (2) the process of melting ferrous scrap in an electric arc furnace (EAF)[26]. The former is the most commonly employed process for steel production in China, accounting for 89% of the total crude steel production in 2019[27]. The major production processes in the ISI involve sintering, pelletizing, coking, ironmaking, steelmaking, and steel rolling [18]. The emission sources include PM emitted by sinter machine heads and tails in the sintering process, pellet firing in the pelletizing process, smokestacks of coke ovens in the coking process, hot stoves and BF casting in the ironmaking process, BOFs or EAFs in the steelmaking process, and reheating furnaces in the steel rolling process.

China enforced stringent emission standards for air pollutants in 2012 (GB 28662-2012, GB 28663-2012, GB 28664-2012, and GB 28665-2012)[14-17], which limited PM emission concentrations originating from sinter machine heads, sinter machine tails, pellet firing, hot stoves, BF castings, BOFs, EAFs, and reheating furnaces to 50, 30, 50, 20, 25, 20, 20, and 20 mg/m^3, respectively. Several emission abatement technologies were applied to meet these emission standards. By the end of 2018, 98.1%, 93.7%, and 17.9% of iron and steel plants (covering 99.0%, 97.8%, and 30.7%, respectively, of the total production processes) had deployed dedusting devices (including electrostatic precipitator (ESP) technology, fabric filter (FF) technology, and wet electrostatic precipitator (WESP) technology), desulfurization devices (including limestone/lime-gypsum wet flue gas desulfurization (WFGD), magnesium oxide WFGD, ammonia-ammonium sulfate WFGD, double alkali WFGD technology, semidry and dry FGD technologies, and pulsed

corona plasma FGD), and denitrification devices (including activated carbon (AC) technology, selective catalytic reduction (SCR) technology, and low-temperature SCR technology) for PM, SO_2, and NO_x removal, respectively. More than 90% of plants had already met the 2015 standards nationwide by the end of 2018[18]. However, most of these plants (81.1%) did not meet the requirements of the ULE standards[18]. After the successive implementation of increasingly stringent standards, the PM emissions resulting from the three processes (i.e., sintering, ironmaking, and BOF-based steelmaking) dominated the total PM emissions of the ISI.

Field measurements and sample collections were conducted at typical units of iron and steel plants located in northern, eastern, and southern China (Supplementary Figure 1). The studied units were selected via comprehensive consideration of the production processes, plant density, control technology options, and plant distribution. The tested units covered provinces featuring substantially high proportions of equipped capacitors and crude steel production (such as Hebei, Jiangsu, Shandong, Shanxi, Guangdong, and Shanghai)[26, 28], encompassing the highest proportions of national plants and 56% of the total crude steel production nationwide in 2019[29]. The sampling units in this study involved all iron and steel production processes (i.e., sintering, ironmaking, steelmaking, and steel rolling) and included the main air pollutant control technology options (i.e., ESP and/or FF, Sd-FGD or WFGD, and selective noncatalytic reduction (SNCR) and/or SCR technologies for PM, SO_2, and NO_x emission mitigation purposes, respectively). The selected production processes, air pollution control devices, and locations of the investigated units are summarized in Supplementary Table 1. The tested units and collected samples sufficiently represented the present real-world PM emissions of the ISI.

3. Studied Power Plant Units

To investigate the emission characteristics and toxic potencies of the PM emissions of power-generating units, field sampling at typical units of coal-, biomass-, and solid waste-fired power plants was conducted in 12 provinces in northern, eastern, southern, and southwestern China from 2017−2022. The electricity generation capacity of the tested units ranged from 9 to 1000 MW, representing the current situation of Chinese power plants. The tested coal-fired units were equipped with pulverized coal boilers or circulating fluidized bed boilers. Bituminous coal, the dominant type of solid fuel among Chinese power and heating plants, was burned in these boilers during the field sampling period. The tested units combined several distinct configurations of

various ULE technology options in this study. All tested units were equipped with SCR and/or SNCR technology to reduce NO_x emissions, a dedusting system (including ESP, FF, and WESP technologies) to reduce PM emissions, and an FGD system (including limestone-gypsum WFGD, seawater WFGD, and semidry FGD technologies) to reduce SO_2 emissions. Wet and semidry FGD technology options were combined with WESP and FF systems as the end-pipe control system to meet the ULE standards. Information on these tested systems is detailed in Supplementary Table 2. The stack inlets after the installed control devices were selected as the sampling sites. Three successful measurements were conducted for each sampling location/process combination. Each tested power unit was operated at a stable generation load of over 75% of its capacity during the field measurements.

4. Studied Cement Units

The cement industry encompassed a total of 1527 facilities operating nationwide in 2018 in mainland China[25]. The major associated production processes of the cement industry include raw material preparation, clinker calcination, and cement grinding[24]. The PM emission sources include clinker production (including quarrying, crushing, and calcination in cement kilns), cement grinding, and fugitive emissions. Three types of kilns, including precalciner kilns, shaft kilns, and other rotary kilns, are commonly used for raw material burning into clinker. To meet the emission standards, a series of control measures were widely employed by the cement industry. Various types of dedicated devices (including cyclones, wet scrubbers, ESPs, and FFs) and denitrification devices (including SNCR and/or SCR devices) were installed in the cement industry for PM and NO_x emission removal, respectively. The FF has currently become the dominant dedicated technology for existing units due to its high removal efficiency, accounting for 86.0% of the total equipped dedicated devices in 2019. SNCR technology is the dominant technology for NO_x emission removal, accounting for 99.0% of the total denitrification devices in 2019. Traditional high-polluting shaft kilns have been gradually replaced by new precalciner kilns with a capacity greater than 4000 t clinker/d, accounting for 97% of the total kilns in 2015[24]. The other two types could be neglected. Precalciner kiln emissions are the largest contributor to PM emissions of the cement industry, accounting for 80% of the total PM emissions in 2015. PM emissions originating from shaft kilns, other rotary kilns, and cement grinding could be neglected. Nearly 83.5% of the cement industry met the new 2015 standards by the end of 2018[25]. Cement units are mainly located

in developing regions of China (including Anhui, Shandong, Sichuan, and Henan provinces)[30], which have introduced local ULE standards to limit the PM concentration to 10 mg/Nm^3 since 2020[25, 30, 31].

Field measurements were conducted at typical units of cement plants equipped with new dry process production lines, which were selected according to the plant proportion, clinker production, air pollution control technology options, and plant distribution. The selected units covered provinces exhibiting substantially high shares of cement plants, clinker and cement production, and air pollutant emissions (such as Hebei, Shandong, Henan, Anhui, Sichuan, Yunnan, and Guangxi provinces)[26]. The covered provinces encompassed 82% of national cement plants[25] and 55% (833.9 Mt) of national total clinker production in 2019[30]. All the tested units were equipped with precalciner kilns, dominating the kilns in the cement industry in China. The production capacity of the test units ranged from 2000 to 12000 t clinker/day. The air pollution control devices installed in the tested units included the dominant devices across China, including FF, WFGD, and SNCR and/or SCR systems. The various combinations and configurations of the tested units could suitably represent the current cement industry across China. Information on the tested units is detailed in Supplementary Table 3. The sampling sites were located at the stack inlets of the kiln heads and kiln tails, where flue gases are directly discharged into the atmosphere. Three successful measurements were conducted for each sampling location/process combination. Each unit was operated at a stable generation load of over 75% of its capacity during the field measurements.

5. Sampling Setup and Procedures

The in-stack sampling method, such as International Organization for Standardization (ISO) 23210:2009[32], Environmental Protection Agency (EPA) Method 5[33], Method 17[34], Method 201A[35], Method 202[36], has been officially recommended by United States EPA and ISO for determining PM emissions from stationary sources (see Supplementary Table 18). Such method has been widely utilized in previous studies to determine PM emissions from various sources, including ultralow emission coal-fired power plants[37, 38], iron and steel industry[39, 40], and cement plants[41]. Hence, a cyclone-based in-stack sampling system (C-5000, ESC, USA) was deployed to separate and collect $PM_{2.5}$ samples from industrial flue gases, following US EPA Method 201A and Method 17. The sampling system, widely used for PM sample collection from stationary sources[42-44], comprises metering consoles, isokinetic sampling nozzles and probes, stainless steel filter holder, umbilical

cables, sample cases, glassware and accessories, vacuum pump, and suspension rail system. To prevent water vapor and fuel gas condensation, the isokinetic sampling nozzle and probe and stainless-steel filter holder were all heated to and maintained at 120 °C throughout the entire sampling process. The PM sampling process was used to assure further analysis and quality control. $PM_{2.5}$ samples were collected on both quartz fiber and Teflon filters for further analysis. Any $PM_{2.5}$ remaining on the filter holders was removed via rinsing twice with acetone and collected in clean glassware for further weighing. The $PM_{2.5}$ sampling time at the power plants, iron and steel plants, and cement plants ranged from 30 to 120 min. Three successful tests were conducted at each sampling site under the same operational conditions. More detailed sampling information can be found in our previous studies[42, 43, 45].

6. Chemicals and Toxicity Analysis and Quality Control

Carbonaceous material, including organic carbon (OC) and elemental carbon (EC), was quantified by using a multiple wavelength thermal/optical instrument (DRI Model 2015, Magee, USA) following the IMPROVE A method. Eight temperature stages were designed to analyze OC1 to OC4 and EC1 to EC3 (i.e., OC fractions at 140, 280, 480, and 580 °C; EC fractions at 580, 740, and 840 °C). Three parallel samples retrieved from each filter were examined. The total carbon on the blank filters was below 0.2 μg C/filter. Organic matter was estimated as 1.2-fold of OC[46]. The analytical procedure has been described in detail elsewhere[47-49]. Blank correction was also performed under the same conditions.

Water-soluble inorganic ions (WSIs) in the $PM_{2.5}$ samples were determined via an ion chromatography (940 Professional IC, Metrohm, Switzerland). One-quarter of each filter was extracted with 10 mL ultrapure water (Millipore, UK) and sonicated for 40 min. C6-150/4.0 and Supp 5-150/4.0 columns (Metrohm, Switzerland) were employed to analyze the cations (Li^+, Na^+, NH_4^+, K^+, Mg^{2+}, and Ca^{2+}) and anions (F^-, Cl^-, Br^-, NO_3^-, PO_4^{3-}, and SO_4^{2-}), respectively, contained in the $PM_{2.5}$ samples. The method detection limits (MDLs) for the ions ranged from 0.47 to 3.33 μg/L. Blank correction was also performed under the same conditions.

Inductively coupled plasma–mass spectrometry (7500a, Thermo Scientific, USA) was employed to quantify the trace elements contained in the $PM_{2.5}$ samples, including Al, Ca, K, Mg, Na, P, S, Si, Li, Be, Sc, Ti, V, Cr, Mn, Fe, Co, Ni, Cu, Zn, Ga, Ge, As, Se, Rb, Sr, Mo, Pd, Ag, Cd, Sn, Sb, Cs, Ba, Pt, Au, Ti, and Pb. The $PM_{2.5}$ samples were digested in Teflon tubes containing 10

mL HNO_3 and 1 mL H_2O_2 and finally diluted to 10 mL with ultrapure water. Filter and procedure blanks were also measured for each analysis process. The recovery rate ranged from 83% to 118%. The relative mass fraction of determined chemical component (i.e., OC, EC, WSIs, elements) is shown in Supplementary Fig. 4 and Supplementary Table 4.

One-quarter of the sampled quartz filters was extracted with dichloromethane via an accelerated solvent extractor (ASE 350, Thermo Scientific, USA), and the extracts were then evaporated, purified, and finally concentrated to 1 mL under purified nitrogen. A gas chromatography coupled with mass spectrometry (Thermo Scientific ISQ 7000 GC−MS, USA) was employed to detect the 16 US EPA priority PAHs (including naphthalene, acenaphthylene, acenaphthene, fluorene, phenanthrene, anthracene, fluoranthene, pyrene, benzo[a]anthracene, chrysene, benzo[b]fluoranthene, chrysene, benzo[b]fluoranthene, benzo[k]fluoranthene, benzo[a]pyrene, dibenzo[a,h]anthracene, indeno[1,2,3-cd]pyrene, and benzo[ghi]perylene). The operation conditions have been detailed in previous studies[50, 51]. Blank samples were also analyzed following the same method. The MDL varied between 23.9 and 51.2 μg/L, while the recovery rate ranged from 80% to 110%.

The human airway epithelial A549 cell line was employed for oxidative stress (OS) potency and cytotoxicity (CT) potency analysis. The reactive oxygen species (ROS) were examined with a 2’,7’-dichlorofluorescein diacetate (DCFH-DA) protocol. The cells were incubated with DCFH-DA for 30 min in the dark and then exposed to the PM extracts for 24 h. The fluorescence intensity was quantified with a microplate reader (Varioskan LUX, Thermo Scientific, USA) at 488/525 nm. The CT potency was measured via a 3-(4,5-dimethylthiazol-2-yl)-2,5-diphenyltetrazolium bromide (MTT) assay. After 24 h of exposure, the absorbance was recorded at 570 nm using a microplate reader (Varioskan LUX, Thermo Scientific, USA).

The endpoint of OS potency ($EC_{1.5}$) was estimated as follows:

$$IR=\frac{\text{Fluorescence intensity (sample)}}{\text{Fluorescence intensity (control)}},$$

$$IR = 1 + \text{slope} \times \text{concentration},$$

$$EC_{1.5}=\frac{0.5}{\text{slope}},$$

where IR indicate the ROS induction ratio of the sample relative to the control. Liner concentration-effect curves with an intercept of 1 and a fitted slope were used to determine the effect concentration at a ROS induction ratio of 1.5 ($EC_{1.5}$).

The contribution of tested metals and PAHs to overall $PM_{2.5}$-induced oxidative stress potency

was estimated based on concentration additional model[52]. Percent concentration = BEQ_{chem}/BEQ_{PM}, $BEQ_{chem}=\sum_{i=1}^{n}(C_i REP_i)$, $REP_i = EC_{1.5,\ t\text{-}BHQ}/EC_{1.5,\ i}$, $BEQ_{PM} = EC_{1.5,\ t\text{-}BHQ}/EC_{1.5,\ PM}$, where BEQ_{chem} is the equivalent concentration of the each identified compound, BEQ_{PM} is the equivalent concentration of PM sample extract estimated against that of t-BHQ as the reference compound, REP_i is the relative effect potency of each identified chemical (i) for ROS generation estimated against that of the reference compound t-BHQ. The additional experimental analysis process and estimation method have been detailed in our previous studies[49, 51].

7. Toxic Components for Industrial Emitted $PM_{2.5}$ Grouped by Processes and Fuel type

The mass-normalized concentrations of $PM_{2.5}$-bound metals from the ISI plants successively increased with their major production processes of sinter ore (224 ± 102 mg/g), pig iron (266 ± 100 mg/g), and crude steel (311 ± 109 mg/g) (Supplementary Fig. 5a). The concentrations of metals per unit mass of $PM_{2.5}$ emitted from power plants ranged from 7.62 to 26.7 mg/g with an averaged values of 16.0 ± 5.1 mg/g for coal-fired units, 11.9 ± 2.9 mg/g for biomass-fired units, and 13.9 ± 3.1 mg/g for solid waste-fired units, as shown in Supplementary Fig. 5a. For the cement production processes, the toxic metals per unit mass of $PM_{2.5}$ emitted from kiln head and kiln tail are 31.0 ± 6.4 mg/g and 19.5 ± 5.1 mg/g, respectively (Supplementary Fig. 5a).

The relative contents of 16 PAHs in ISI-emitted $PM_{2.5}$ varied with the major production processes of sinter ore, pig iron, and crude steel. The PAHs emitted from BF-based ironmaking process (976 ± 889 μg/g) was nearly 2.7 times that of sinter machine-emitted PAHs and 2.8 times that of BOF-emitted PAHs (Supplementary Fig. 5b). The mass-normalized concentration of $PM_{2.5}$-bound PAHs emission from solid waste-fired plants (36.1 ± 52.4 μg/g) was approximately 2.4 times that of CFPP-emitted PAHs and 1.2 times that of biomass-fired plants-emitted PAHs (Supplementary Fig. 5b). While the concentration for kiln tail-emitted PAHs (17.2 ± 15.4 μg/g) was nearly 1.8-fold higher than that for kiln head-emitted PAHs in the cement industry (Supplementary Fig. 5b).

8. Model Simulation Performance

In this study, the latest version of the Community Multiscale Air Quality (CMAQ) model (version 5.3.3) was employed to simulate the concentration of air pollutants. Version 6 of the carbon bond mechanism (CB6) was adopted for the gas-phase chemistry, and AERO7 was employed as the

aerosol module[53]. The meteorological fields were simulated with the Weather Research and Forecasting (WRF) model (version 3.9.1). The modeling domain and WRF modeling options were the same as those used in our previous study[3]. The horizontal resolution was 27 km × 27 km, and the vertical extent was divided into 14 layers. The updated Air Benefit and Cost and Attainment Assessment System (ABaCAS) emission inventory combined with the WRF-CMAQ model was employed to simulate PM mass concentrations in China. Regarding the emission inventory for other countries/regions in the modeling domain, we used the emission inventory of IIASA-ECLIPSE (version 5, base year 2015).

The ABaCAS emission inventory, which was comprehensively evaluated from 2005 to 2017 in previous studies[3, 54-57], was adopted to evaluate the emission inventory for 2019 with a modeling system. The hourly modeled $PM_{2.5}$ concentration was compared to observation data of the China National Environmental Monitoring Centre (Supplementary Table 12). We used the normalized mean bias (NMB), normalized mean error (NME), mean fractional bias (MFB), and mean fractional error (MFE) to quantitatively evaluate the model performance[58]. The model slightly underestimated the concentration (-8%), but the MFB and MFE values for all seasons satisfied the performance criteria. In general, the modeling system and emission inventory for 2019 could be used to reproduce the pollution level in China in 2019.

9. Estimation of Toxic Units and Toxic Equivalent Values

Toxic units (TUs), including estimation of $PM_{2.5}$-induced OS and CT potency values, can be estimated as follows: $TUOS_{j,s} = \frac{1}{(EC_{1.5})_{j,s}}$ and $TUCT_{j,s} = \frac{1}{(IC_{20})_{j,s}}$, where j and s denote sample (j) and industry source (s), respectively, $EC_{1.5}$ is the endpoint of the $PM_{2.5}$-induced OS potency, indicating the concentration causing a 1.5-fold increase in intracellular ROS formation relative to that in the control group, IC_{20} is the endpoint of the $PM_{2.5}$-induced CT potency, indicating the concentration causing 20% inhibition of the cell viability relative to that in the control group. Region-specific TUs for iron and steel, power, and cement plants were estimated at the provincial level. The average provincial TUs of industrial $PM_{2.5}$ emissions can be estimated as follows: $TUOS_{i,s} = \frac{1}{\sum(EC_{1.5})_{j,s} \times WF_{j,s}}$ and $TUCT_{i,s} = \frac{1}{\sum(IC_{20})_{j,s} \times WF_{j,s}}$, where WF is the weighing factor, estimated based on the mass fraction of $PM_{2.5}$ emissions of each individual manufacturing process (or each individual fuel-fired unit) to the total $PM_{2.5}$ emission amount of all manufacturing

processes (or all fuel-fired units). The same TU values were adopted for the cement plants across China because the toxic potency of the $PM_{2.5}$ emissions of cement plants slightly varied in the different regions. Toxic equivalent values were estimated based on the TUs. An OS equivalent value of 1 was adopted for the cement plants (the lowest endpoint of the industrial $PM_{2.5}$-induced OS), and the relative OS equivalent values for other industrial sectors can be estimated as $\frac{(\mathrm{TUOS_s})_i}{(\mathrm{TUOS_{CP}})_i}$ $\times$ $\mathrm{TEOS_{CP}}$, where $(\mathrm{TUOS}_s)_i$ is the OS-related TU for industry source *s* in each geographic unit (*i*) and $(\mathrm{TUOS}_{CP})_i$ is the OS TU for the cement plants in each geographic unit (*i*). A CT equivalent value of 1 was employed for the CFPPs (the lowest endpoint of the industrial $PM_{2.5}$-induced CT), and the relative CT equivalent values for other industrial sources can be estimated as $\frac{(\mathrm{TUCT_s})_i}{(\mathrm{TUCT_{CFPP}})_i}$ $\times$ $\mathrm{TECT_{CFPP}}$ (Supplementary Table 6). Toxic potency-adjusted emissions were estimated based on $PM_{2.5}$ emissions and $PM_{2.5}$-associated toxic equivalent values.

10. Unit Cost of Implementing the ULE standards for PM control

The unit cost of the end-of-pipe measures for primary PM emissions was calculated for the different types of end-of-pipe technology options in each sector. The initial investment cost and fixed operating and maintenance cost (*FOM*) were collected from government documents, reports, literature review, and field investigation, as described in our previous study[59]. *CC* and *FOM* data of the various emission control technology options at CFPPs, iron and steel production plants, and cement production plants are summarized in Supplementary Tables 14–16. Cost data for ULE retrofitting of power plants, the ISI, and the cement industry were updated in this study by collecting the most recent information from government and local documents and reports. According to a survey of the Ministry of Ecology and Environment (MEE) of China[60], regarding ULE retrofitting of current power plants, the initial investment for PM control measures is approximately 56.5 CNY/kW. The *FOM* level for PM control measures is approximately 0.0099 CNY/kWh. Regarding ULE retrofitting in the ISI, data were collected from surveys[61] and a report of Hebei province (environmental impact report of the joint restructuring and urban steel mill relocation and transformation project of Hebei Zongheng Group Fengnan Iron and Steel Co., http://info.hebei.gov.cn/hbszfxxgk/329975/329988/330110/ 6772689/index.html), as detailed in Supplementary Table 17. Regarding ULE retrofitting for PM control purposes, the *TCost* value is approximately 29.12 CNY/t steel. In regard to ULE retrofitting for PM control purposes in the

cement industry, the initial investment for high-efficiency fabric filters is 12 and 15 million CNY at plants with production capacities of 2500 and 5000 tons cement clinker per day, respectively[59].

11. Uncertainty analysis

Uncertainty was a crucial consideration in this study, covering all aspects from the $PM_{2.5}$ emission factors (EFs) to the risk index for the population-weighted TPAE. The uncertainty analysis of $PM_{2.5}$ EFs, chemical components, and $PM_{2.5}$-related toxic potency were evaluated based on the standard deviation (SD) derived from the analysis results of various parallel samples. The uncertainties of the emission inventory of each source were quantified with 10000 times Monte Carlo simulation based on the uncertainty range of the activity data, EFs, end-of-pipe control efficiencies, the same as our previous study[62].

The uncertainties of the toxic potency adjusted-emissions were quantified based on the SDs of the $PM_{2.5}$-related toxic potency and $PM_{2.5}$ emission. As for the population-weighted TPAE, we limited our uncertainty analysis to the emission inventory owing to the near-linear response of the ambient $PM_{2.5}$ concentration to the primary $PM_{2.5}$ emission. The uncertainties inherent in the chemical transport model itself were not taken into account as they are a form of systematic error and are directly tied to the model selection. Moreover, the model employed in this study has undergone model evaluation and meets the model performance criteria.

Supplementary Figures

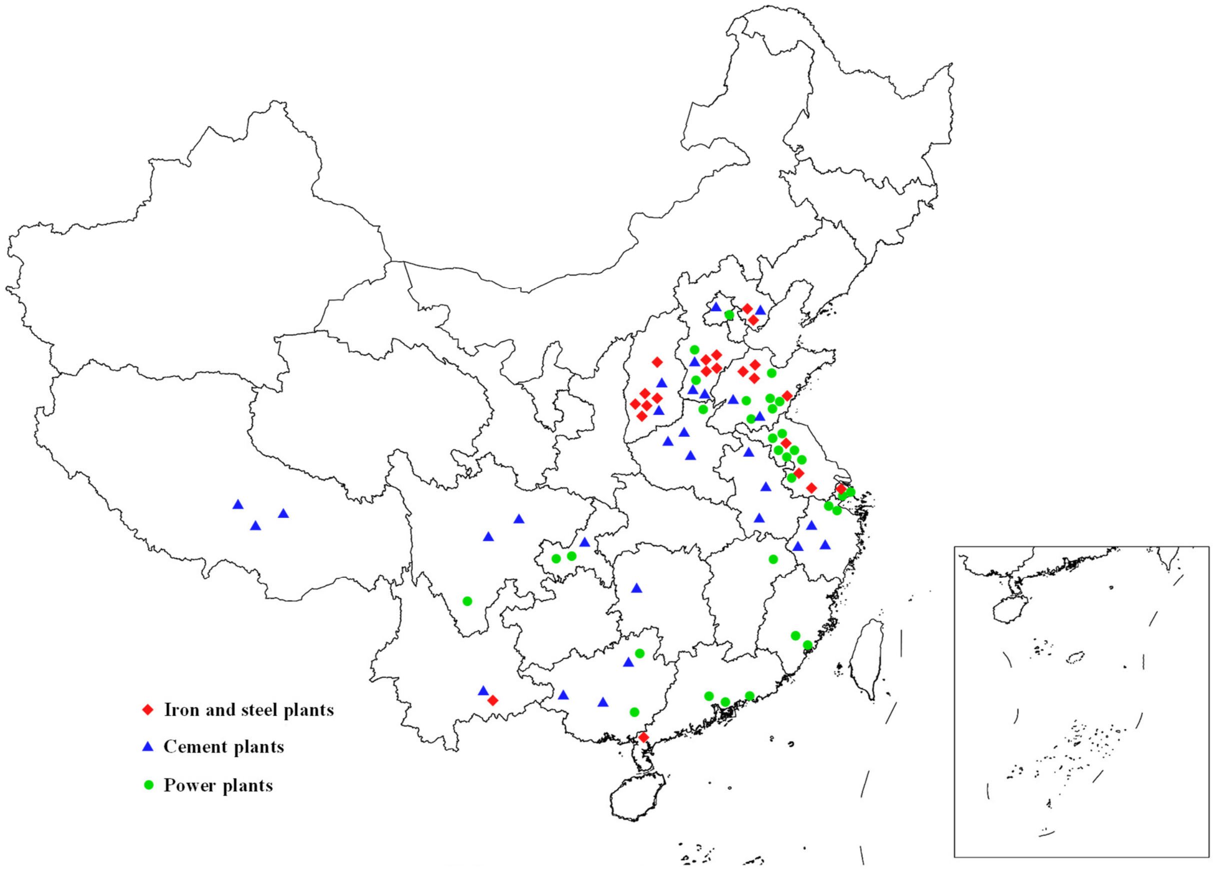

Supplementary Figure 1. Geographic distribution of the field-studied industrial plants in mainland China. The red diamonds, blue triangles, and green circles indicate iron and steel plants, cement plants, and power plants, respectively. The map was provided by NCAR Command Language (NCL)[63].

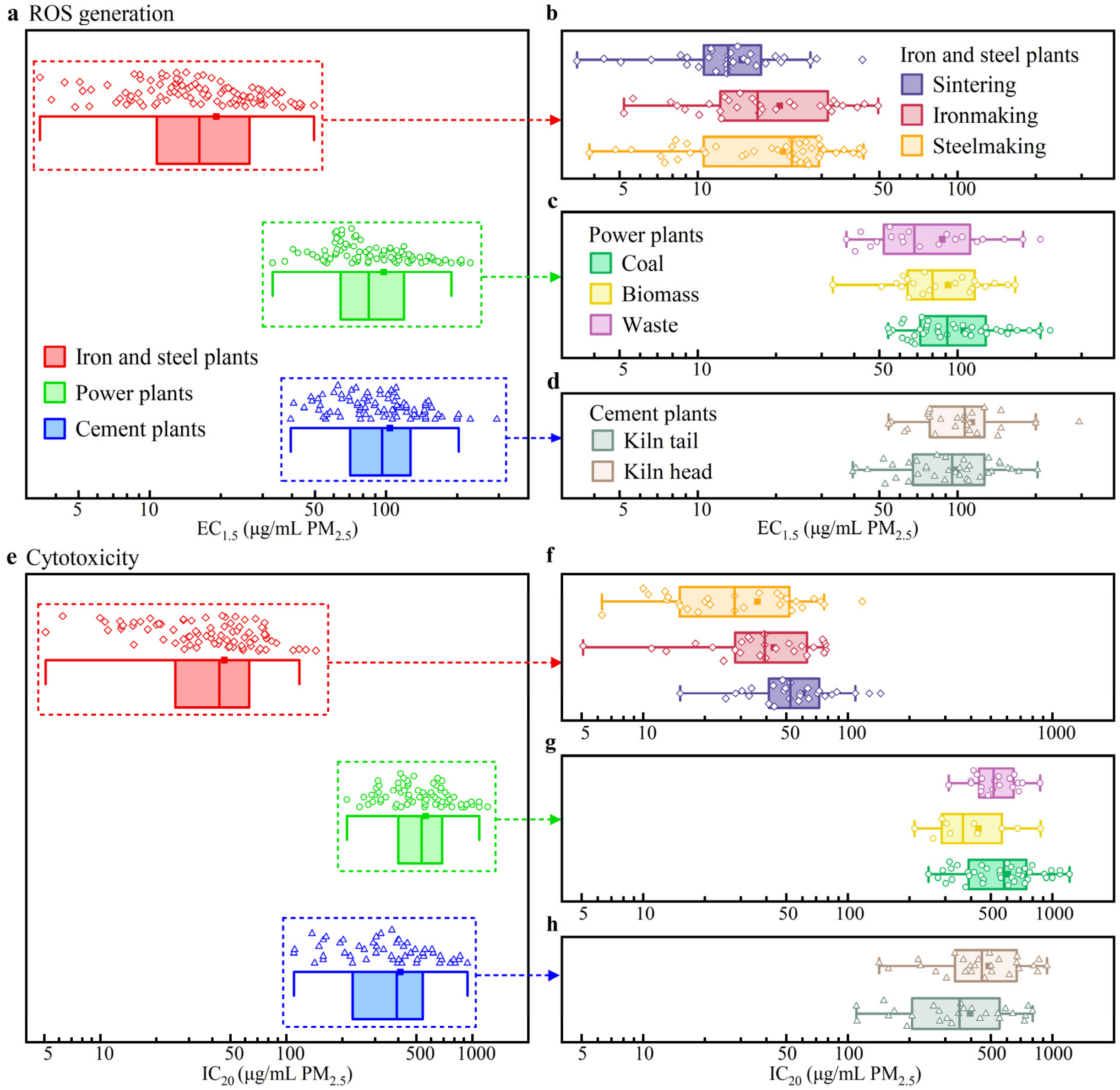

Supplementary Figure 2. Toxic potencies of the sampled PM2.5 originating from the three industrial sectors. PM2.5-induced oxidative stress potency (**a**) and cytotoxicity potency (**e**) of iron and steel plants, power plants, and cement plants and those grouped by the prevailing iron and steel production processes (including sintering, ironmaking, and basic oxygen furnace (BOF)-based steelmaking) (**b**) and (**f**), fuel type of power-generating units (including coal, biomass, and solid waste) (**c**) and (**g**), and prevailing clinker and cement production processes (including kiln head and kiln tail) (**d**) and (**h**).

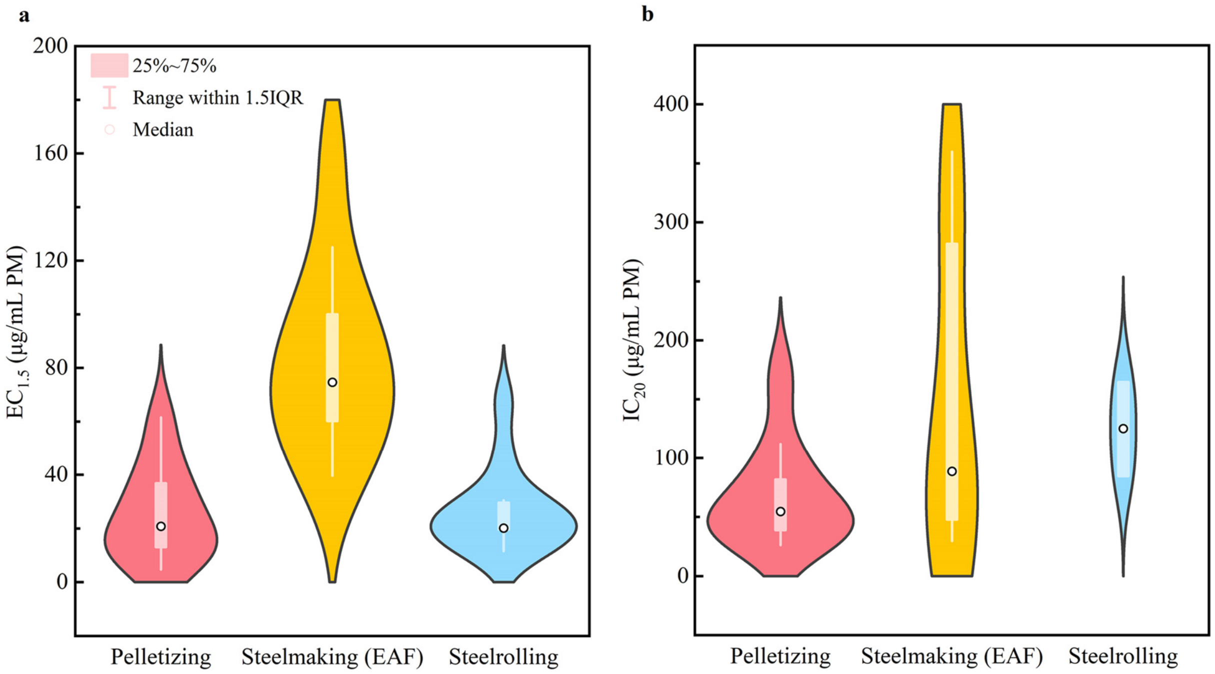

Supplementary Figure 3. Oxidative stress potencies (**a**) and cytotoxicity potencies (**b**) induced by $PM_{2.5}$ emitted from typical iron and steel industrial processes, including pelletizing, electric arc furnace (EAF)-based steelmaking, and steel rolling processes. These processes are major processes categorized as other processes of the iron and steel sector, whose major processes include sintering, ironmaking, and basic oxygen furnace (BOF)-based steelmaking.

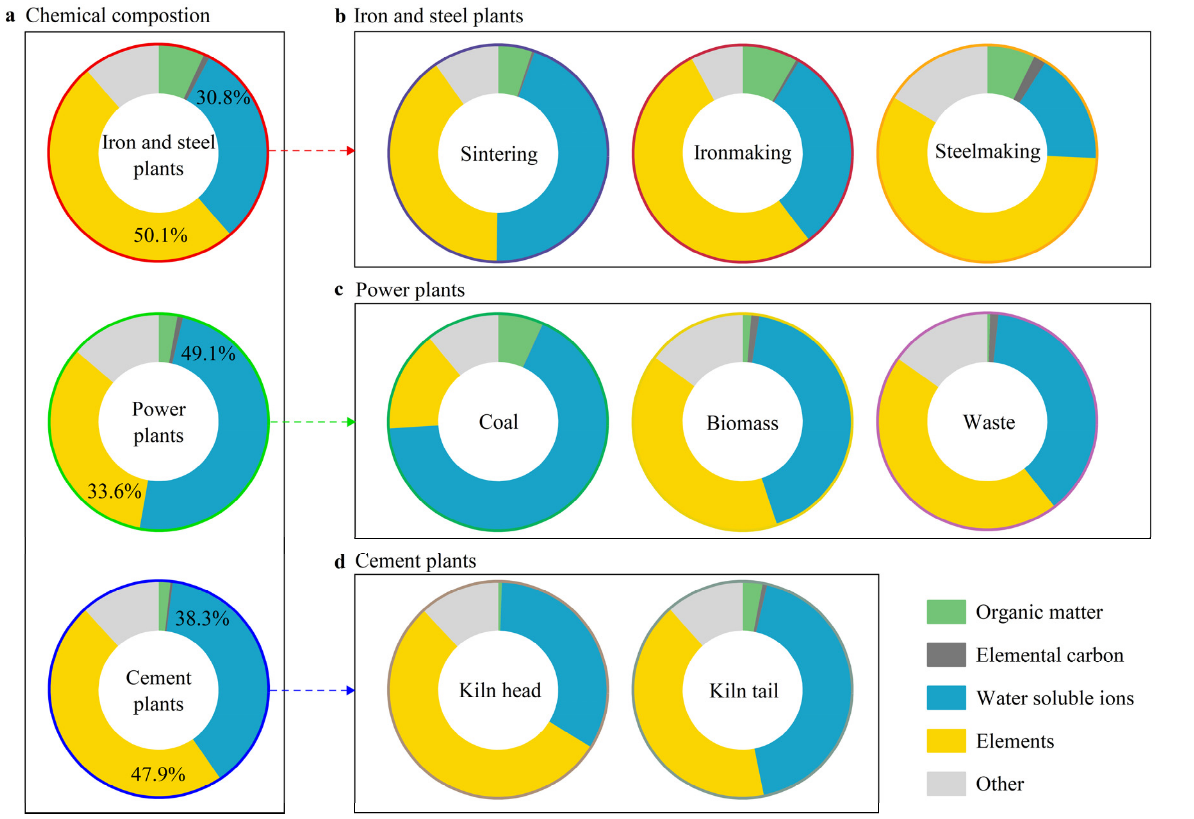

Supplementary Figure 4. Relative mass fractions of the chemical components in $PM_{2.5}$ emitted from iron and steel plants, power plants, and cement plants (**a**) and those grouped by the prevailing iron and steel production processes (including sintering, ironmaking, and basic oxygen furnace (BOF)-based steelmaking) (**b**), fuel type of power-generating units (including coal, biomass, and solid waste) (**c**), and prevailing clinker and cement production processes (including kiln head and kiln tail) (**d**). The organic matter content is estimated as 1.2 times the organic carbon content; the water-soluble inorganic ions include anions (F^-, Cl^-, Br^-, NO_3^-, PO_4^{3-}, and SO_4^{2-}) and cations (Li^+, Na^+, NH_4^+, K^+, Ca^{2+}, and Mg^{2+}); the elements include Li, Be, Al, Si, P, S, K, Ca, Sc, Ti, V, Cr, Mn, Fe, Co, Ni, Cu, Zn, Ga, Ge, As, Se, Rb, Sr, Mo, Pd, Ag, Cd, Sn, Sb, Cs, Ba, Pt, Au, TI, and Pb. Other indicates undermined species.

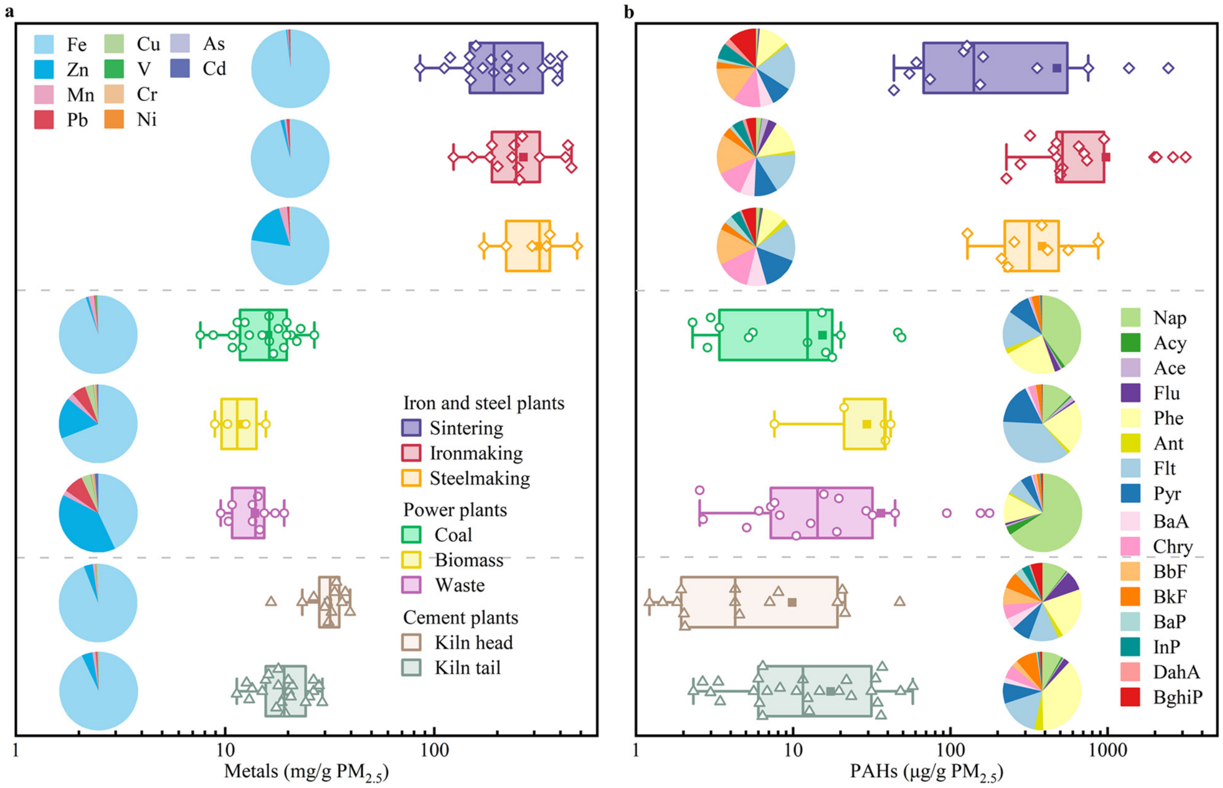

Supplementary Figure 5. Mass-normalized concentrations of (**a**) the 10 selected toxic metals (i.e., V, Cr, Mn, Fe, Ni, Cu, Zn, As, Cd, and Pb) and (**b**) the 16 US EPA priority PAHs (i.e., Nap, Acy, Ace, Flu, Phe, Ant, Flt, Pyr, BaA, Chry, BbF, BkF, BaP, InP, DahA, Bghip) per unit mass of $PM_{2.5}$ originating from the field-studied units at iron and steel, power, and cement plants. The tested units in the three industrial sectors were grouped by the crude steel production processes (i.e., sintering, blast furnace-based ironmaking, and BOF-based steelmaking), fuel type (i.e., coal, biomass, and solid waste), and clinker production processes (i.e., kiln head and kiln tail).

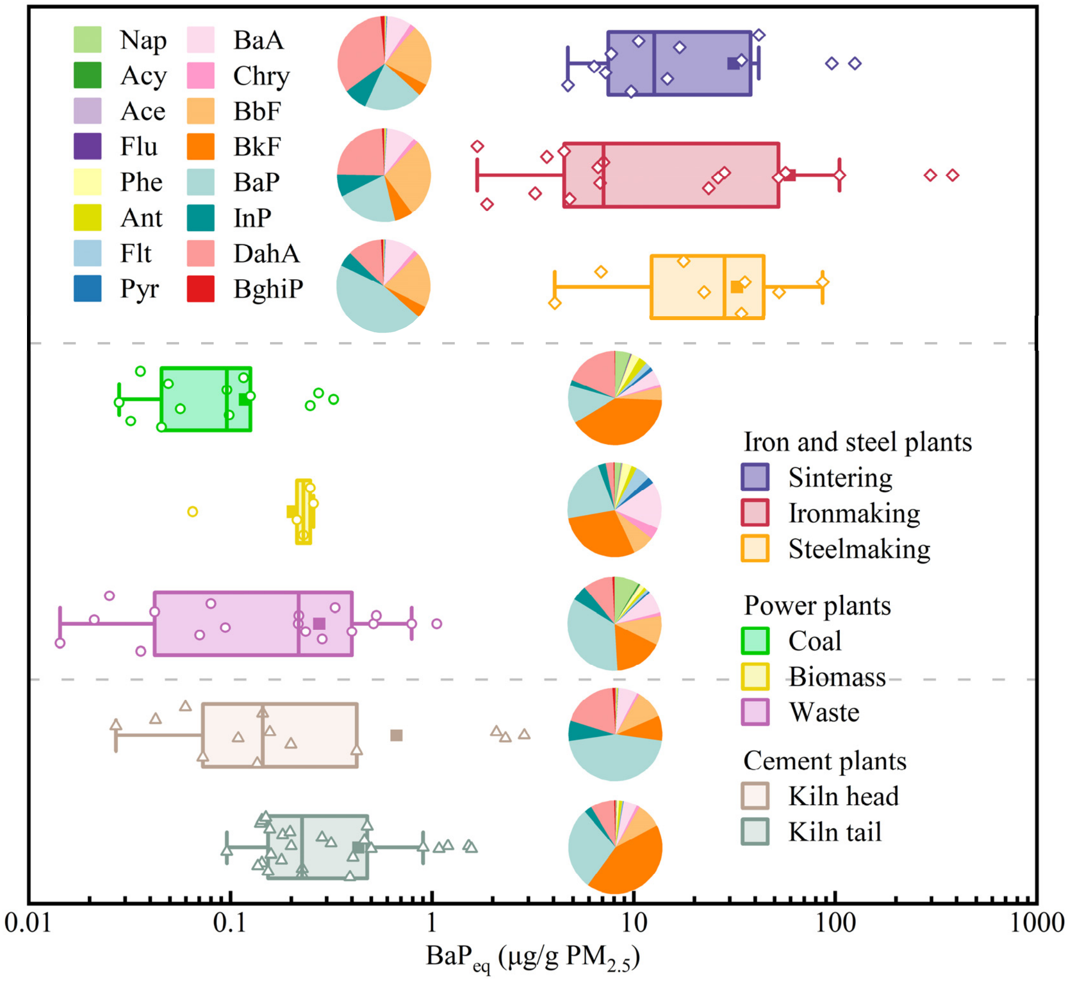

Supplementary Figure 6. Mass-normalized BaP_{eq} values of $PM_{2.5}$ originating from iron and steel, power, and cement plants. BaP_{eq} values, estimated to determine the carcinogenic risk of the 16 US EPA priority PAHs, were calculated by considering the toxic equivalent factors of each PAH species.

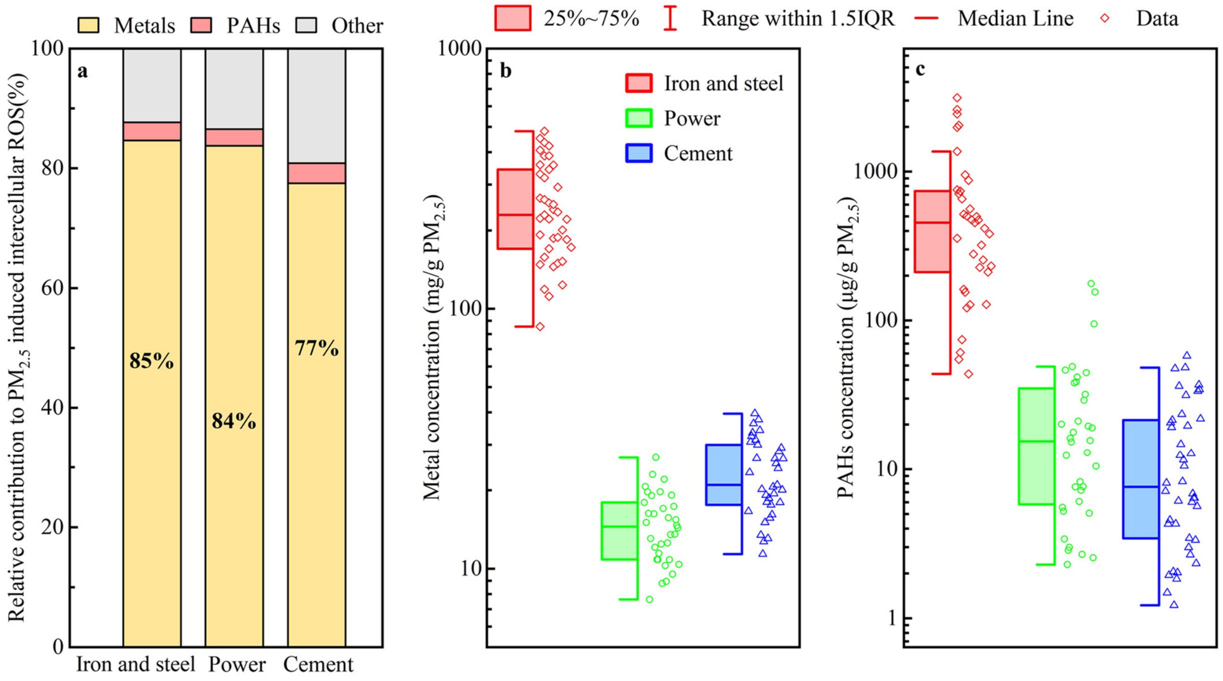

Supplementary Figure 7. Relative fractional contributions of the targeted 10 toxic metals (i.e., V, Cr, Mn, Fe, Ni, Cu, Zn, As, Cd, and Pb) and 16 US EPA priority control PAHs (i.e., Nap, Acy, Ace, Flu, Phe, Ant, Flt, Pyr, BaA, Chry, BbF, BkF, BaP, InP, DahA, and Bghip) to the oxidative stress potency induced by $PM_{2.5}$ emissions from iron and steel, power, and cement plants (**a**). The concentrations of the targeted 10 toxic metals (**b**) and 16 US EPA priority control PAHs (**c**) per unit mass of $PM_{2.5}$ originating from the three industrial plants. The quantitative contributions of each individual toxic species were estimated based on the chemical addition reference model[52].

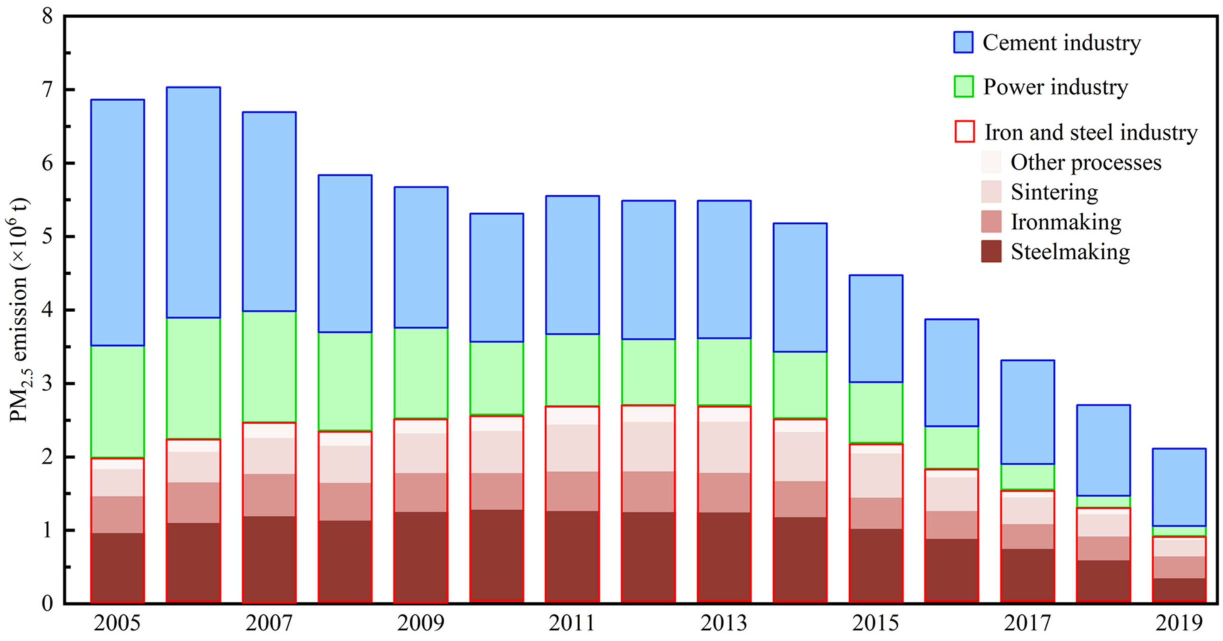

Supplementary Figure 8. Temporal changes in $PM_{2.5}$ emissions from the three studied industrial sectors (i.e., iron and steel industry, power industry, and cement industry) in mainland China. The emissions of the iron and steel industry can be grouped by major production processes (i.e., sintering, blast furnace-based ironmaking, and basic oxygen furnace-based steelmaking) and other processes (i.e., pelletizing, electric arc furnace-based steelmaking, and steel rolling).

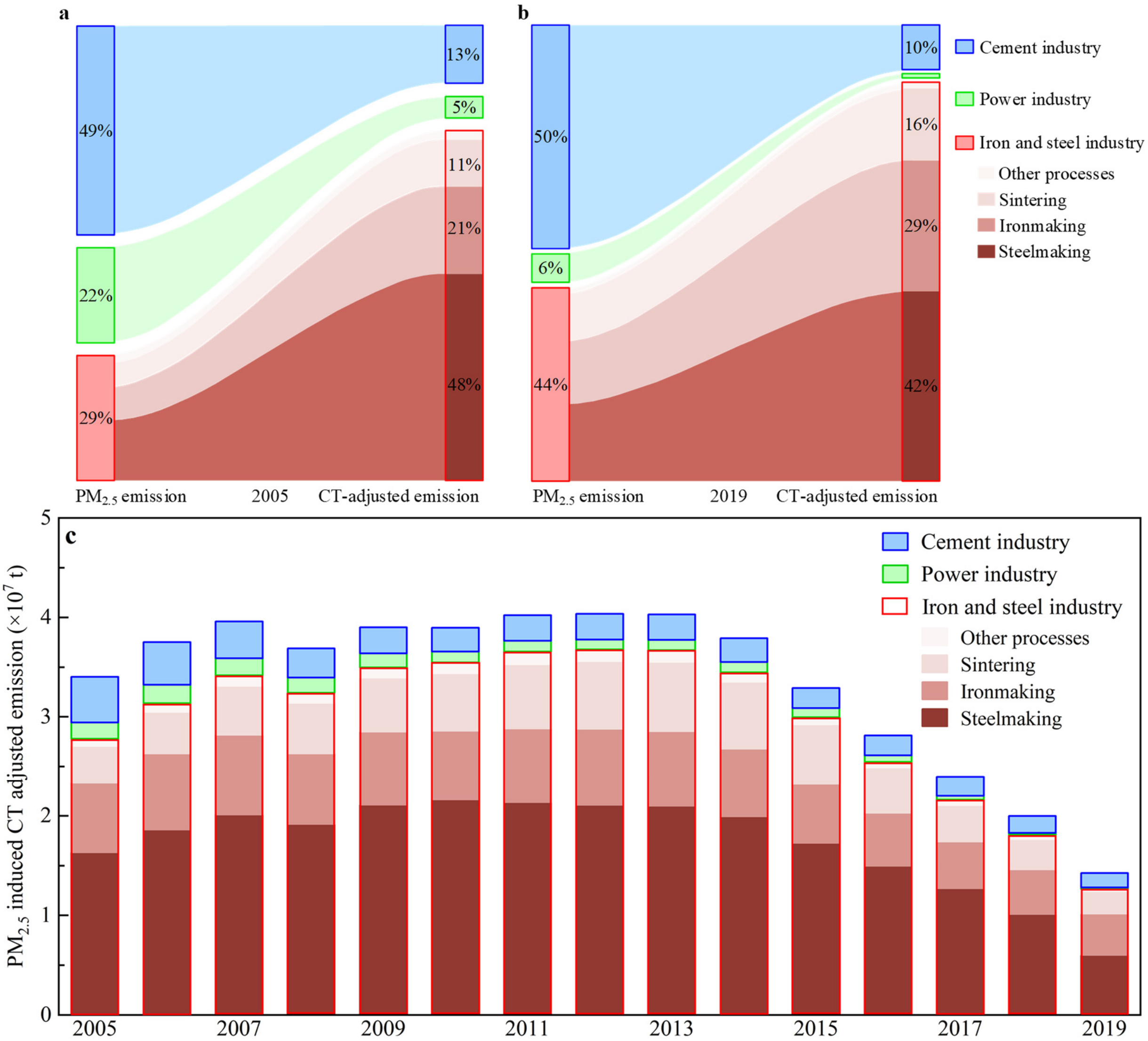

Supplementary Figure 9. Changes in $PM_{2.5}$ emissions and $PM_{2.5}$-induced CT-adjusted emissions from industrial sectors in China. $PM_{2.5}$ emissions and CT-adjusted emissions in 2005 (**a**) and 2019 (**b**). Total $PM_{2.5}$-induced cytotoxicity-adjusted emissions and the corresponding changes related to variances in iron and steel production grouped by process (including sintering, ironmaking, basic oxygen furnace-based steelmaking, and other process), power generation, and cement production between 2005 and 2019 (**c**).

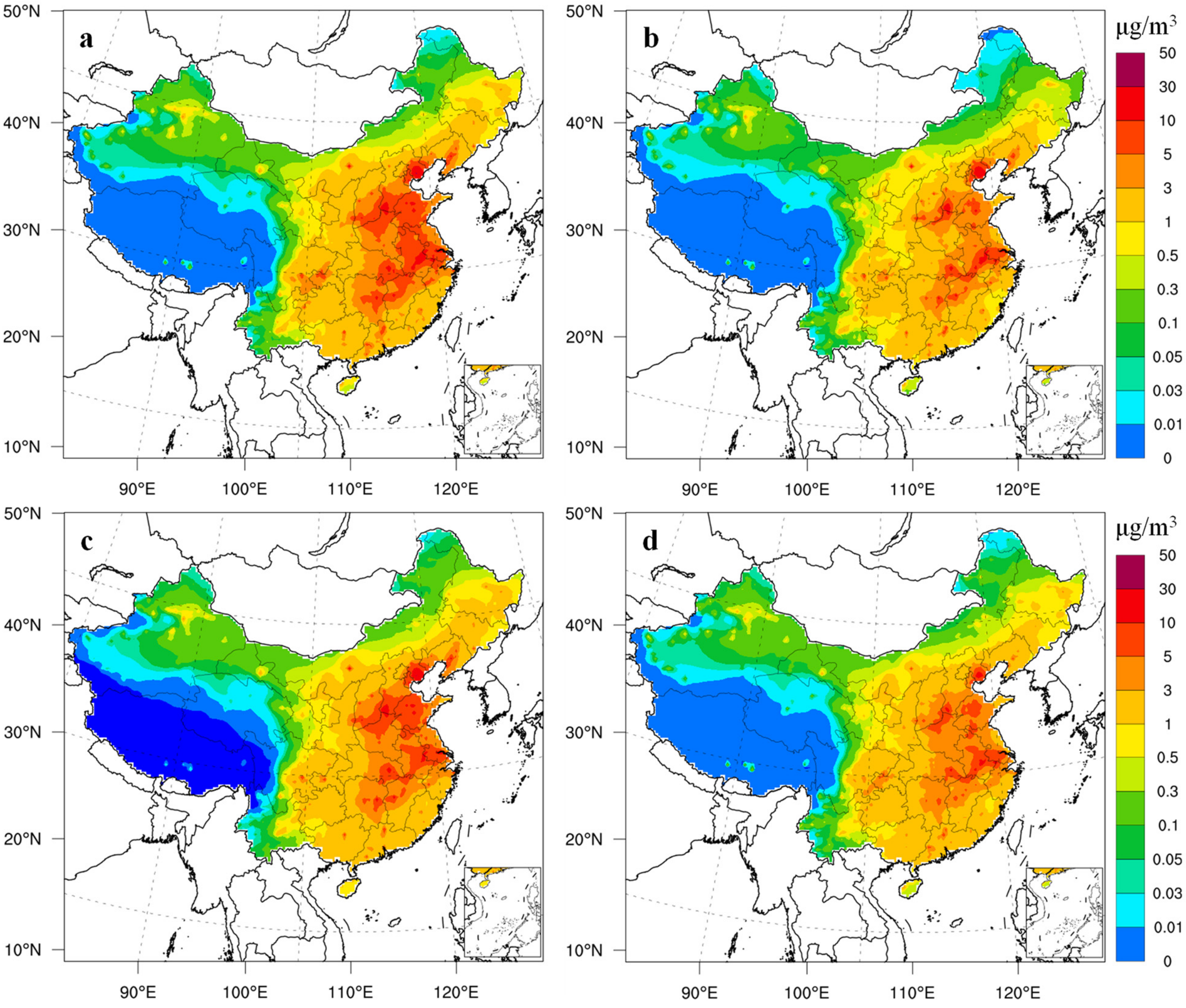

Supplementary Figure 10. Spatial distribution of the total ambient $PM_{2.5}$ concentration originating from the three industrial sectors before (**a**) and after the implementation of the ULE standards in the power industry (**b**), cement industry (**c**), and iron and steel industry (**d**) in 2019, respectively. The maps were provided by NCAR Command Language (NCL)[63].

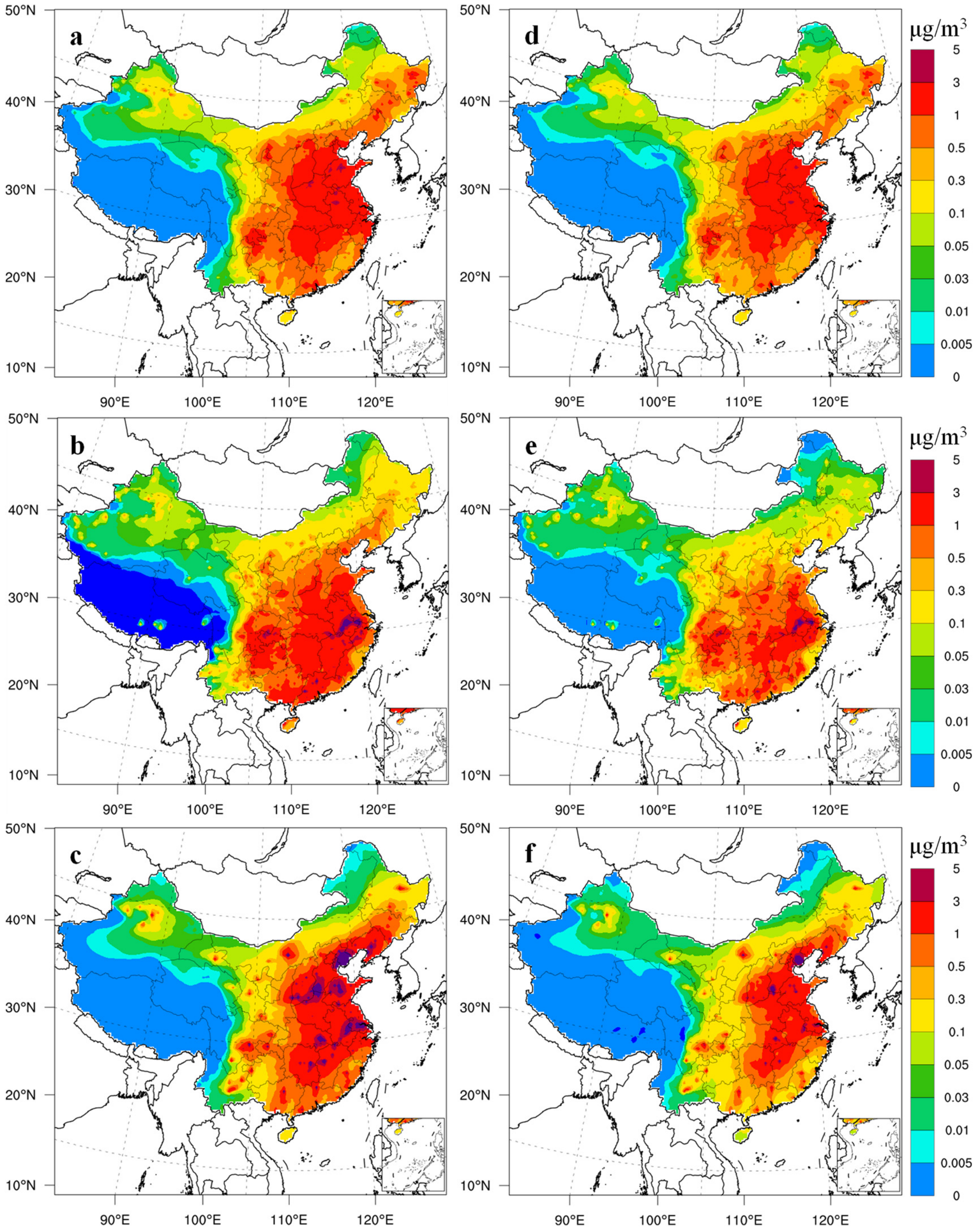

Supplementary Figure 11. Spatial distribution of the ambient $PM_{2.5}$ concentration originating from the power industry (**a**), cement industry (**b**), and iron and steel industry (**c**) before the implementation of the ULE standards and the reduction in the ambient $PM_{2.5}$ concentration attributed to the implementation of the ULE standards in the power industry (**d**), cement industry (**e**), and iron and steel industry (**f**) in 2019, respectively. The maps were provided by NCAR Command Language (NCL)[63].

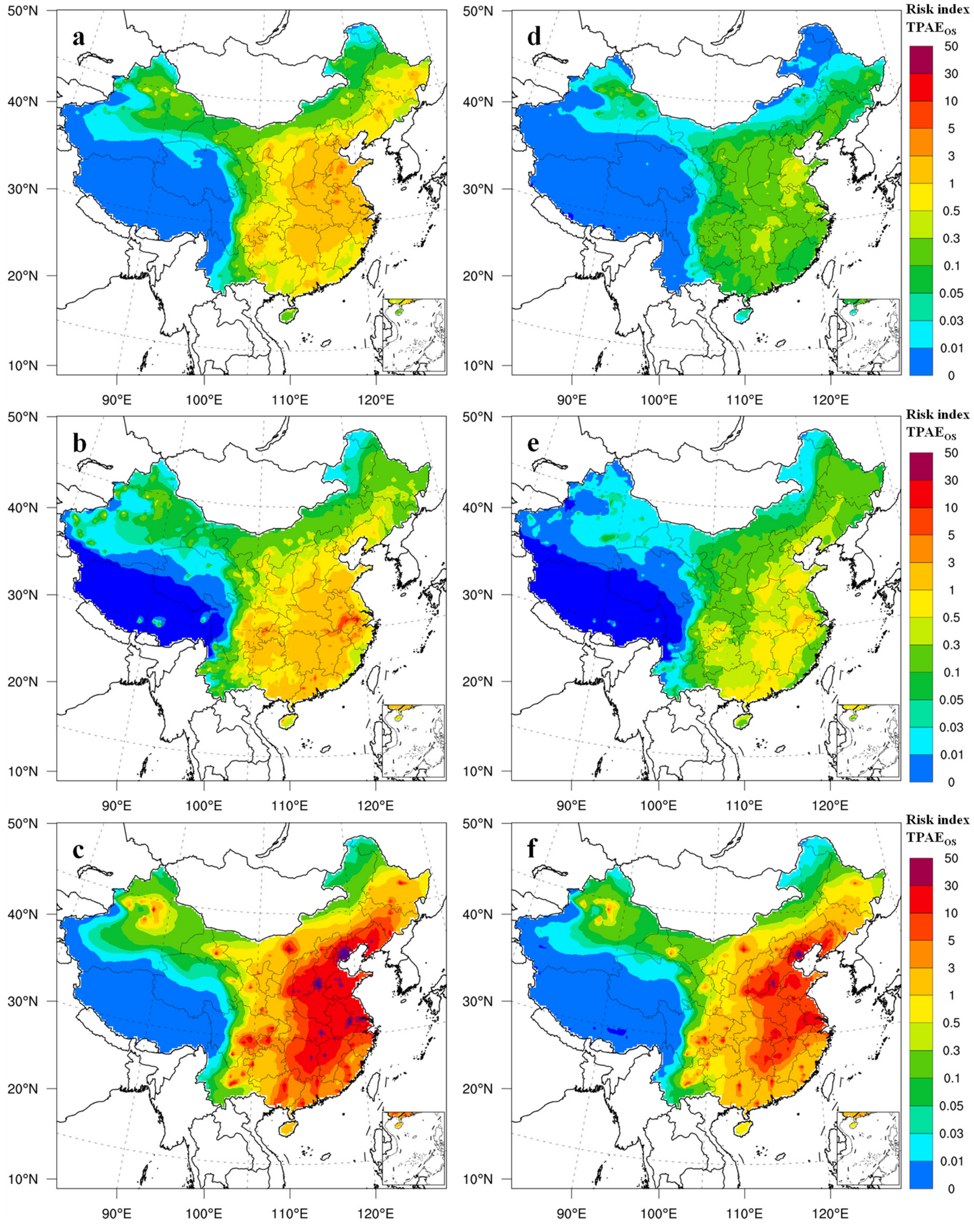

Supplementary Figure 12. Spatial distribution of TPAE$_{OS}$ originating from the power industry, cement industry, and iron and steel industry before (**a-c**) and after (**d-f**) the implementation of the ULE standards in 2019, respectively. The maps were provided by NCAR Command Language (NCL)[63].

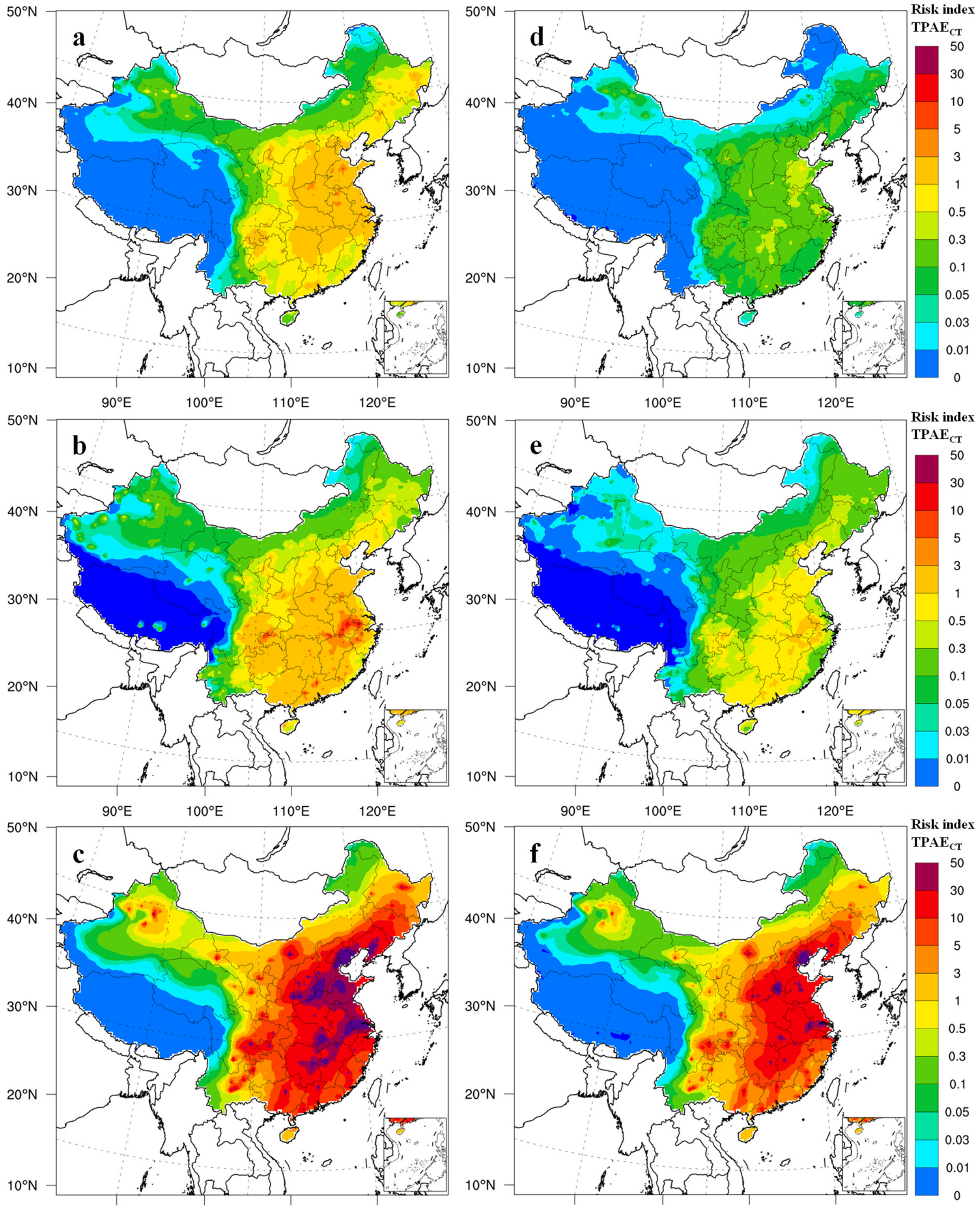

Supplementary Figure 13. Spatial distribution of $TPAE_{CT}$ originating from the power industry, cement industry, and iron and steel industry before (**a**-**c**) and after (**d**-**f**) the implementation of the ULE standards in 2019, respectively. The maps were provided by NCAR Command Language (NCL)[63].

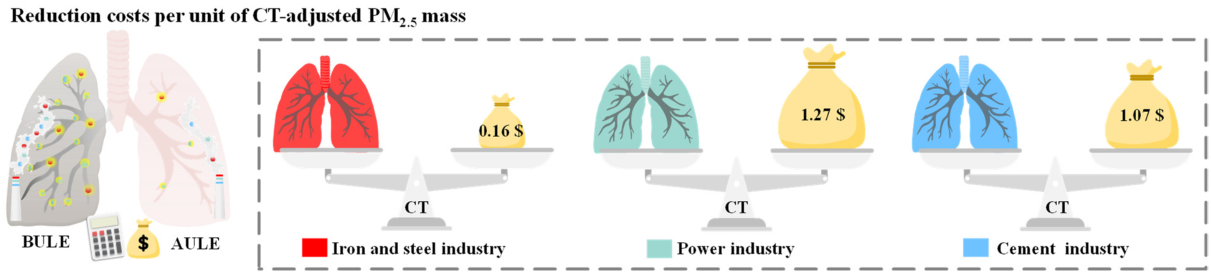

Supplementary Figure 14. Reduction cost per ton of $PM_{2.5}$-induced cytotoxicity (CT) potency-adjusted $PM_{2.5}$ mass when meeting the ULE standards in the iron and steel industry (red), power industry (green), and cement industry (blue).

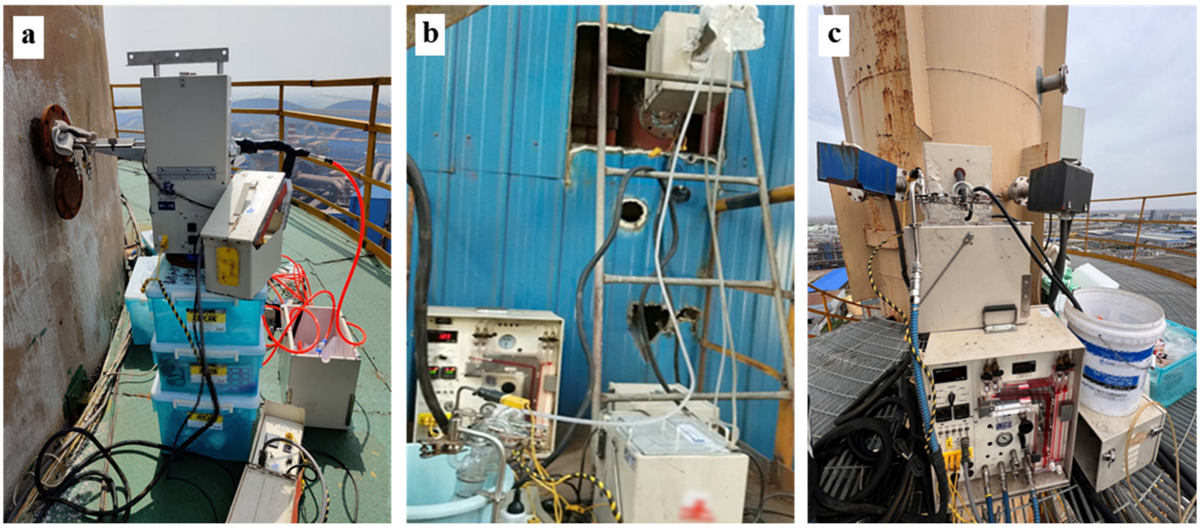

Supplementary Figure 15. Photographs of the sampling systems employed at the typical stack inlets in the iron and steel industry (**a**), power industry (**b**), and cement industry (**c**).

Supplementary Tables

Supplementary Table 1. Information on the sampling units of the studied iron and steel plants and corresponding air pollution control devices (APCDs).

Unit	Process	Equipment	APCD	Location
ISP-1	Iron making	Blast furnace	ESP+FF	Hebei
	Steel making	Electric arc furnace	ESP+FF	
ISP-2	Iron making	Blast furnace	ESP+FF	Hebei
	Steel making	Converter	ESP+FF	
ISP-3	Sintering	Sinter machine	ESP+SCR+FF	Hebei
ISP-4	Sintering	Sinter machine	ESP+SCR+FF	Hebei
ISP-5	Sintering	Sinter machine	ESP+SCR+FF	Hebei
ISP-6	Sintering	Sinter machine	ESP+SCR+FF	Hebei
ISP-7	Sintering	Sinter machine	SCR+ESP+Sd-FGD	Shanxi
	Iron making	Blast furnace	FF	
	Steel making	Converter	FF	
ISP-8	Sintering	Sinter machine	SCR+WFGD+WESP	Shanxi
	Iron making	Blast furnace	FF	
ISP-9	Sintering	Sinter machine	ESP+FF+AC-FGD	Shanxi
	Iron making	Blast furnace	FF	
	Steel making	Converter	ESP+FF	
	Steel rolling	Reheating furnace	LNB+SCR+FF	
ISP-10	Coking	Coke oven	FF+Sd-FGD+SCR	Shanxi
ISP-11	Coking	Coke oven	FF+Sd-FGD+SCR	Shanxi
ISP-12	Sintering	Sinter machine	AC	Shanxi
ISP-13	Sintering	Sinter machine	ESP+CFB-FGD+SCR+FF	Shanghai
	Iron making	Blast furnace	FF	
ISP-14	Sintering	Sinter machine	ESP+COA+FF/ESP	Jiangsu
ISP-15	Sintering	Sinter machine	Sd-FGD+FF/SCR	Jiangsu
	Iron making	Blast furnace	FF	
	Steel making	Converter	FF	
	Steel rolling	Reheating furnace	FF	
ISP-16	Steel rolling	Heating furnace	FF	Jiangsu
ISP-17	Pelletizing	Roasting machine	SNCR-SCR+ESP+WFGD+WESP	Shandong
ISP-18	Pelletizing	Roasting machine	SNCR-SCR+ESP+WFGD+WESP	Shandong
ISP-19	Steel making	Electric arc furnace	FF	Shandong
ISP-20	Sintering	Sinter machine	ESP+L-WFGD+WESP	Shandong
ISP-21	Sintering	Sinter machine	ESP+AC	Guangdong
	Iron making	Blast furnace	ESP+FF	
	Steel making	Converter	ESP+FF	
ISP-22	Iron making	Blast furnace	FF	Yunnan

Notes: APCD, air pollution control device;

ESP, electrostatic precipitator; WESP, wet electrostatic precipitator; AC, activated coke; FF, fabric filter;

LNB, low-NO_x boiler; SCR, selective catalytic reduction; SNCR, selective noncatalytic reduction;

Sd-FGD, semidry flue gas desulfurization; AC-FGD, activated coke flue gas desulfurization; COA: circulating oxidation absorption; CFB-FGD, circulating fluidized bed flue gas desulfurization; L-WFGD, limestone-gypsum wet flue gas desulfurization.

Supplementary Table 2. Information on the sampling units of the studied power plants and corresponding air pollution control devices (APCDs).

Unit	Capacity (MW)	Boiler type	Fuel type	APCD	Location
PP-1	660	PCB	Bituminite	SCR+ESP+L-WFGD+WESP	Hebei
PP-2	760	PCB	Bituminite	LNB+SCR+ESP+L-WFGD+WESP	Hebei
PP-3	2×350	SFB	Bituminite	LNB+SCR+ESP+L-WFGD+WESP	Hebei
PP-4	1000	PCB	Bituminite	SCR+ESP+L-WFGD+WESP	Shanghai
PP-5	30	Incinerator	Others	SNCR+AC+FF+SCR	Shanghai
PP-6	20	PCB	Bituminite	SCR+FF+A-WFGD+WSEP	Jiangsu
PP-7	20	PCB	Bituminite	SCR+FF+A-WFGD+WSEP	Jiangsu
PP-8	20	PCB	Bituminite	SCR+FF+A-WFGD+WSEP	Jiangsu
PP-9	3×22.5	PCB	Bituminite	SNCR+SCR+FF+L-WFGD	Jiangsu
PP-10	30	BB	Biomass	SNCR+PNCR+Sd-FGD+FF	Jiangsu
PP-11	12	BB	Biomass	FGD+FF	Jiangsu
PP-12	12	BB	Biomass	L-FGD+FF	Jiangsu
PP-13	600	PCB	Bituminite	SCR+ESP+L-WFGD+WESP	Zhejiang
PP-14	1000	PCB	Bituminite	SCR+ESP+L-WFGD+WESP	Zhejiang
PP-15	9	Incinerator	Others	AC+FF	Fujian
PP-16	40	Incinerator	Others	AC+FF	Fujian
PP-17	12	Incinerator	Others	SNCR+Sd-FGD+AC+D-FGD+FF	Jiangxi
PP-18	2×350	PCB	Bituminite	SCR+ESP+S-WFGD+WESP	Shandong
PP-19	2×680	PCB	Bituminite	SCR+ESP+S-WFGD+WESP	Shandong
PP-20	2×25	CFB	Bituminite	SCR+ESP+L-WFGD+WESP	Shandong
PP-21	2×350	CFB	Bituminite	SCR+ESP+L-WFGD+WESP	Shandong
PP-22	135	CFB	Bituminite	SCR+FF+Sd-FGD+WESP	Shandong
PP-23	2×1000	PCB	Bituminite	LNB+SCR+ESP+L-WFGD+WESP	Shandong
PP-24	200	PCB	Bituminite	SCR+ESP+L-WFGD+WESP	Henan
PP-25	2×30	Incinerator	Others	SNCR+AC+FF+SCR	Guangdong
PP-26	15	Incinerator	Others	SNCR+AC+FF	Guangdong
PP-27	2×25	Incinerator	Others	SNCR+AC+FF+SCR	Guangdong
PP-28	27	Incinerator	Others	SNCR+AC+FF	Guangxi
PP-29	35	Incinerator	Others	SNCR+AC+FF	Guangxi
PP-30	18	Incinerator	Others	FF+Sd-FGD	Chongqing
PP-31	24	Incinerator	Others	Sd-FGC+FF	Chongqing
PP-32	12	Incinerator	Others	AC+FF	Sichuan

Notes: APCD, air pollution control device;

PCB, pulverized coal-fired boiler; BB, biomass boiler; CFB, circulating fluidized bed; LNB, low-NO_x boiler; SCR, selective catalytic reduction; SNCR, selective noncatalytic reduction;

ESP, electrostatic precipitator; WESP, wet electrostatic precipitator; AC, activated coke; FF, fabric filter;

L-WFGD, limestone-gypsum wet flue gas desulfurization; A-WFGD, ammonia wet flue gas desulfurization;

S-WFGD, seawater wet flue gas desulfurization; Sd-FGD, semidry flue gas desulfurization; D-FGD, dry flue gas desulfurization.

Supplementary Table 3. Information on the sampling units of the studied cement plants and their air pollution control devices (APCDs).

Unit	Kiln type	Production capacity (ton/day)	APCD	Sampling site	Location
CM-1	NSP	2500	SNCR+FF	Cement kiln	Beijing
CM-2	NSP	4500	SNCR+FF	Cement kiln	Hebei
CM-3	NSP	4500	SNCR+FF	Cement kiln	Hebei
CM-4	NSP	5000	SNCR+FF	Cement kiln	Hebei
CM-5	NSP	2000	SNCR+LNB+FF	Cement kiln	Hebei
CM-6	NSP	3000	FF	Kiln head<br>Kiln tail	Shanxi
CM-7	NSP	5000	SNCR+SCR+FF	Kiln head<br>Kiln tail<br>Coal mill	Zhejiang
CM-8	NSP	5000	SNCR+SCR+FF	Kiln head<br>Kiln tail<br>Coal mill	Zhejiang
CM-9	NSP	5000	SNCR+SCR+FF	Kiln head<br>Kiln tail<br>Coal mill	Zhejiang
CM-10	NSP	5000	SNCR+FF	Cement kiln	Anhui
CM-11	NSP	5000	SNCR+SCR+WFGD+FF	Kiln head<br>Kiln tail	Anhui
CM-12	NSP	5000	SNCR+SCR+WFGD+FF	Kiln head<br>Kiln tail<br>Coal mill	Anhui
CM-13	NSP	4000	FF	Cement kiln	Shandong
CM-14	NSP	4000	FF	Cement kiln	Shandong
CM-15	NSP	12000	SNCR+FF	Cement kiln	Henan
CM-16	NSP	4500	FF	Cement kiln	Henan
CM-17	NSP	4500	SNCR+WFGD+FF	Cement kiln	Henan
CM-18	NSP	5000	SNCR+SCR+FF	Kiln head<br>Kiln tail<br>Coal mill	Hunan
CM-19	NSP	4000	SNCR+FF	Kiln head<br>Kiln tail<br>Coal mill	Guangxi
CM-20	NSP	4000	SNCR+FF	Kiln head<br>Kiln tail<br>Coal mill	Guangxi
CM-21	NSP	4000	SNCR+FF	Kiln head<br>Kiln tail<br>Coal mill	Guangxi
CM-22	NSP	4500	SNCR+WFGD+FF	Kiln head<br>Kiln tail	Chongqing

				Coal mill	
CM-23	NSP	4500	SNCR+WFGD+FF	Kiln head<br>Kiln tail<br>Coal mill	Sichuan
CM-24	NSP	4500	SNCR+WFGD+FF	Kiln head<br>Kiln tail<br>Coal mill	Sichuan
CM-25	NSP	2500	SNCR+FF	Cement kiln	Yunnan
CM-26	NSP	2500	FF<br>SNCR+FF<br>FF	Kiln head<br>Kiln tail<br>Coal mill	Tibet
CM-27	NSP	2500	FF<br>SNCR+FF<br>FF	Kiln head<br>Kiln tail<br>Coal mill	Tibet
CM-28	NSP	2500	FF<br>SNCR+FF<br>FF	Kiln head<br>Kiln tail<br>Coal mill	Tibet

Notes: NSP, new suspension preheater;

SNCR, selective noncatalytic reduction; FF, fabric filter;

LNB, low-NO_x boiler; SCR, selective catalytic reduction;

WFGD, wet flue gas desulfurization.

Supplementary Table 4. Chemical composition and toxic potency of the $PM_{2.5}$ emissions from the iron and steel industry, power industry, and cement industry.

Industrial sector	OC (%)	EC (%)	WSI (%)	Sulfate (%)	Nitrate (%)	Element (%)	$EC_{1.5}$ (μg/mL)	IC_{20} (μg/mL)
Iron and steel	5.71 ± 1.33	0.90 ± 0.82	30.8 ± 14.1	9.71 ± 8.34	0.15 ± 0.20	50.1 ± 9.2	19.1 ± 10.9	46.4 ± 28.2
Power	2.33 ± 2.16	0.84 ± 0.69	49.1 ± 15.9	12.6 ± 11.2	0.88 ± 1.19	33.6 ± 16.2	94.9 ± 41.9	561 ± 226
Cement	1.74 ± 1.68	0.34 ± 0.48	38.3 ± 7.1	8.03 ± 3.91	10.7 ± 2.1	47.9 ± 9.0	103.6 ± 46.3	442 ± 221

Supplementary Table 5. Population-weighted exposure to $PM_{2.5}$ (μg/m^3) originating from the power industry (PI), cement industry (CI), and iron and steel industry (ISI) at the provincial level with or without meeting the ultralow emission standards in 2019.

Region	No ultralow emission			Under ultralow emission		
	PI	CI	ISI	PI	CI	ISI
China	2.04 ± 0.45	1.256 ± 0.039	1.13 ± 0.30	0.99 ± 0.22	0.173 ± 0.005	0.38 ± 0.10
Beijing	1.90 ± 0.42	1.012 ± 0.031	0.54 ± 0.14	0.95 ± 0.21	0.139 ± 0.004	0.31 ± 0.08
Tianjin	6.42 ± 1.42	1.571 ± 0.048	0.65 ± 0.17	3.23 ± 0.72	0.195 ± 0.006	0.37 ± 0.10
Hebei	5.34 ± 1.19	1.550 ± 0.048	0.76 ± 0.20	2.64 ± 0.59	0.19 ± 0.006	0.35 ± 0.09
Shanxi	2.59 ± 0.57	1.541 ± 0.048	0.76 ± 0.20	1.25 ± 0.28	0.172 ± 0.005	0.28 ± 0.07
Inner Mongolia	1.19 ± 0.26	0.463 ± 0.014	0.29 ± 0.08	0.53 ± 0.12	0.061 ± 0.002	0.12 ± 0.03
Liaoning	3.14 ± 0.70	0.731 ± 0.023	0.61 ± 0.16	1.47 ± 0.33	0.124 ± 0.004	0.34 ± 0.09
Jilin	0.85 ± 0.19	0.754 ± 0.023	0.45 ± 0.12	0.39 ± 0.09	0.091 ± 0.003	0.25 ± 0.07
Heilongjiang	0.33 ± 0.07	0.689 ± 0.021	0.31 ± 0.08	0.14 ± 0.03	0.057 ± 0.002	0.20 ± 0.05
Shanghai	3.23 ± 0.72	1.827 ± 0.056	0.47 ± 0.12	1.86 ± 0.41	0.273 ± 0.008	0.16 ± 0.04
Jiangsu	4.16 ± 0.92	1.981 ± 0.061	1.32 ± 0.35	2.15 ± 0.48	0.271 ± 0.008	0.39 ± 0.10
Zhejiang	1.48 ± 0.33	1.723 ± 0.053	1.30 ± 0.34	0.73 ± 0.16	0.191 ± 0.006	0.38 ± 0.10
Anhui	2.93 ± 0.65	2.292 ± 0.071	2.72 ± 0.71	1.44 ± 0.32	0.27 ± 0.008	0.67 ± 0.18
Fujian	0.74 ± 0.16	0.635 ± 0.02	0.59 ± 0.16	0.33 ± 0.07	0.082 ± 0.003	0.17 ± 0.04
Jiangxi	2.32 ± 0.51	1.090 ± 0.034	2.20 ± 0.58	1.10 ± 0.24	0.163 ± 0.005	0.67 ± 0.18
Shandong	3.06 ± 0.68	2.340 ± 0.072	1.21 ± 0.32	1.48 ± 0.33	0.342 ± 0.011	0.49 ± 0.13
Henan	2.64 ± 0.59	2.286 ± 0.071	1.27 ± 0.33	1.31 ± 0.29	0.252 ± 0.008	0.47 ± 0.12
Hubei	4.17 ± 0.93	1.755 ± 0.054	1.70 ± 0.45	1.92 ± 0.43	0.265 ± 0.008	0.50 ± 0.13
Hunan	2.05 ± 0.45	1.453 ± 0.045	1.67 ± 0.44	0.95 ± 0.21	0.290 ± 0.009	0.51 ± 0.13
Guangdong	0.79 ± 0.18	0.896 ± 0.028	1.16 ± 0.30	0.39 ± 0.09	0.138 ± 0.004	0.50 ± 0.13
Guangxi	0.94 ± 0.21	0.518 ± 0.016	1.17 ± 0.31	0.42 ± 0.09	0.085 ± 0.003	0.45 ± 0.12
Hainan	0.21 ± 0.05	0.279 ± 0.009	0.66 ± 0.17	0.11 ± 0.02	0.035 ± 0.001	0.38 ± 0.10
Chongqing	0.90 ± 0.20	0.871 ± 0.027	2.17 ± 0.57	0.40 ± 0.09	0.15 ± 0.005	0.50 ± 0.13
Sichuan	0.70 ± 0.16	0.660 ± 0.020	1.25 ± 0.33	0.33 ± 0.07	0.097 ± 0.003	0.31 ± 0.08
Guizhou	0.43 ± 0.10	0.756 ± 0.023	1.28 ± 0.34	0.20 ± 0.04	0.104 ± 0.003	0.36 ± 0.09
Yunnan	0.23 ± 0.05	0.182 ± 0.006	0.39 ± 0.10	0.10 ± 0.02	0.026 ± 0.001	0.10 ± 0.03
Tibet	0 ± 0	0 ± 0	0 ± 0	0 ± 0	0 ± 0	0 ± 0
Shaanxi	0.64 ± 0.14	0.739 ± 0.023	0.74 ± 0.19	0.31 ± 0.07	0.143 ± 0.004	0.19 ± 0.05
Gansu	0.27 ± 0.06	0.262 ± 0.008	0.36 ± 0.09	0.12 ± 0.03	0.045 ± 0.001	0.08 ± 0.02
Qinghai	0.19 ± 0.04	0.088 ± 0.003	0.23 ± 0.06	0.08 ± 0.02	0.021 ± 0.001	0.04 ± 0.01
Ningxia	0.30 ± 0.07	0.752 ± 0.023	0.65 ± 0.17	0.13 ± 0.03	0.147 ± 0.005	0.13 ± 0.03
Xinjiang	0.40 ± 0.09	0.090 ± 0.003	0.11 ± 0.03	0.17 ± 0.04	0.029 ± 0.001	0.02 ± 0.01

Supplementary Table 6. Toxic equivalent values of the $PM_{2.5}$-related oxidative stress (OS) and cytotoxicity (CT) potencies of the iron and steel industry (ISI), power industry (PI), and cement industry (CI) at the provincial level in 2019.

Region	ISI		PI		CI	
	OS	CT	OS	CT	OS	CT
China	6.21 ± 3.40	13.69 ± 8.50	1.11 ± 0.47	1.05 ± 0.45	1.07 ± 0.47	1.37 ± 0.69
Beijing	6.21 ± 3.40	13.69 ± 8.50	1.23 ± 0.48	1.37 ± 0.66	1.07 ± 0.47	1.37 ± 0.69
Tianjin	6.20 ± 3.41	13.91 ± 8.55	1.12 ± 0.47	1.07 ± 0.46	1.07 ± 0.47	1.37 ± 0.69
Hebei	6.14 ± 3.37	13.62 ± 8.32	1.10 ± 0.47	1.03 ± 0.44	1.07 ± 0.47	1.37 ± 0.69
Shanxi	6.10 ± 3.34	13.45 ± 8.17	1.10 ± 0.47	1.02 ± 0.43	1.07 ± 0.47	1.37 ± 0.69
Inner Mongolia	6.22 ± 3.41	13.61 ± 8.52	1.09 ± 0.47	1.01 ± 0.43	1.07 ± 0.47	1.37 ± 0.69
Liaoning	6.22 ± 3.40	13.64 ± 8.49	1.10 ± 0.47	1.01 ± 0.43	1.07 ± 0.47	1.37 ± 0.69
Jilin	6.18 ± 3.39	13.52 ± 8.40	1.12 ± 0.47	1.09 ± 0.48	1.07 ± 0.47	1.37 ± 0.69
Heilongjiang	6.04 ± 3.28	12.89 ± 7.79	1.16 ± 0.47	1.20 ± 0.55	1.07 ± 0.47	1.37 ± 0.69
Shanghai	5.89 ± 3.32	13.56 ± 7.97	1.10 ± 0.47	1.04 ± 0.44	1.07 ± 0.47	1.37 ± 0.69
Jiangsu	6.42 ± 3.51	14.33 ± 9.15	1.10 ± 0.47	1.03 ± 0.44	1.07 ± 0.47	1.37 ± 0.69
Zhejiang	6.16 ± 3.35	13.34 ± 8.23	1.13 ± 0.47	1.10 ± 0.49	1.07 ± 0.47	1.37 ± 0.69
Anhui	6.28 ± 3.44	13.74 ± 8.68	1.11 ± 0.47	1.05 ± 0.46	1.07 ± 0.47	1.37 ± 0.69
Fujian	6.51 ± 3.53	14.06 ± 9.24	1.12 ± 0.47	1.08 ± 0.48	1.07 ± 0.47	1.37 ± 0.69
Jiangxi	6.06 ± 3.32	13.26 ± 8.02	1.11 ± 0.47	1.06 ± 0.46	1.07 ± 0.47	1.37 ± 0.69
Shandong	6.07 ± 3.32	13.33 ± 8.05	1.11 ± 0.47	1.05 ± 0.45	1.07 ± 0.47	1.37 ± 0.69
Henan	6.25 ± 3.42	13.89 ± 8.63	1.12 ± 0.47	1.08 ± 0.47	1.07 ± 0.47	1.37 ± 0.69
Hubei	6.30 ± 3.44	13.78 ± 8.71	1.11 ± 0.47	1.05 ± 0.46	1.07 ± 0.47	1.37 ± 0.69
Hunan	6.24 ± 3.42	13.65 ± 8.56	1.10 ± 0.47	1.04 ± 0.44	1.07 ± 0.47	1.37 ± 0.69
Guangdong	6.33 ± 3.47	14.08 ± 8.93	1.12 ± 0.47	1.06 ± 0.46	1.07 ± 0.47	1.37 ± 0.69
Guangxi	6.45 ± 3.51	14.02 ± 9.09	1.13 ± 0.47	1.09 ± 0.48	1.07 ± 0.47	1.37 ± 0.69
Hainan	6.21 ± 3.40	13.69 ± 8.50	1.18 ± 0.47	1.26 ± 0.59	1.07 ± 0.47	1.37 ± 0.69
Chongqing	6.39 ± 3.48	13.96 ± 8.94	1.11 ± 0.47	1.04 ± 0.45	1.07 ± 0.47	1.37 ± 0.69
Sichuan	6.26 ± 3.43	13.66 ± 8.61	1.13 ± 0.47	1.11 ± 0.49	1.07 ± 0.47	1.37 ± 0.69
Guizhou	6.21 ± 3.40	13.5 ± 8.46	1.10 ± 0.47	1.03 ± 0.44	1.07 ± 0.47	1.37 ± 0.69
Yunnan	6.28 ± 3.43	13.78 ± 8.67	1.11 ± 0.47	1.04 ± 0.45	1.07 ± 0.47	1.37 ± 0.69
Tibet	6.21 ± 3.40	13.69 ± 8.50	1.11 ± 0.47	1.05 ± 0.45	1.07 ± 0.47	1.37 ± 0.69
Shaanxi	6.33 ± 3.46	13.99 ± 8.84	1.09 ± 0.47	1.00 ± 0.42	1.07 ± 0.47	1.37 ± 0.69
Gansu	6.14 ± 3.35	13.2 ± 8.18	1.10 ± 0.47	1.02 ± 0.44	1.07 ± 0.47	1.37 ± 0.69
Qinghai	6.18 ± 3.39	13.44 ± 8.37	1.09 ± 0.47	1.00 ± 0.42	1.07 ± 0.47	1.37 ± 0.69
Ningxia	6.58 ± 3.57	14.24 ± 9.46	1.09 ± 0.47	1.00 ± 0.42	1.07 ± 0.47	1.37 ± 0.69
Xinjiang	6.14 ± 3.37	13.37 ± 8.26	1.09 ± 0.47	1.00 ± 0.42	1.07 ± 0.47	1.37 ± 0.69

Supplementary Table 7. Reduction in the risk index for the population-weighted $TPAE_{OS}$ and $TPAE_{CT}$ attributed to meeting the ULE standards in the power industry (PI), cement industry (CI), and iron and steel industry (ISI) at the provincial level in 2019, respectively.

Region	Reduction in TAPEOS			Reduction in TAPECT		
	PI	CI	ISI	PI	CI	ISI
China	1.20 ± 0.51	0.80 ± 0.44	6.53 ± 3.85	1.14 ± 0.49	1.03 ± 0.58	14.38 ± 9.46
Beijing	1.07 ± 0.42	0.25 ± 0.08	5.91 ± 3.49	1.19 ± 0.58	0.32 ± 0.18	13.01 ± 8.56
Tianjin	1.53 ± 0.65	0.29 ± 0.18	19.77 ± 11.71	1.47 ± 0.64	0.38 ± 0.22	44.36 ± 28.96
Hebei	1.50 ± 0.64	0.44 ± 0.25	16.58 ± 9.80	1.40 ± 0.61	0.56 ± 0.32	36.78 ± 23.88
Shanxi	1.50 ± 0.64	0.51 ± 0.29	8.17 ± 4.83	1.40 ± 0.60	0.66 ± 0.37	18.02 ± 11.64
Inner Mongolia	0.44 ± 0.19	0.18 ± 0.11	4.10 ± 2.42	0.41 ± 0.17	0.24 ± 0.14	8.98 ± 5.96
Liaoning	0.66 ± 0.28	0.29 ± 0.18	10.38 ± 6.13	0.61 ± 0.26	0.36 ± 0.21	22.78 ± 15.04
Jilin	0.75 ± 0.31	0.21 ± 0.12	2.84 ± 1.68	0.72 ± 0.32	0.28 ± 0.15	6.22 ± 4.10
Heilongjiang	0.73 ± 0.30	0.12 ± 0.06	1.14 ± 0.67	0.75 ± 0.35	0.15 ± 0.08	2.45 ± 1.58
Shanghai	1.72 ± 0.73	0.33 ± 0.19	8.07 ± 4.88	1.61 ± 0.69	0.42 ± 0.25	18.58 ± 11.66
Jiangsu	1.89 ± 0.80	0.99 ± 0.55	12.91 ± 7.61	1.77 ± 0.76	1.28 ± 0.73	28.80 ± 19.45
Zhejiang	1.72 ± 0.72	0.98 ± 0.52	4.62 ± 2.71	1.69 ± 0.75	1.26 ± 0.71	10.00 ± 6.55
Anhui	2.25 ± 0.95	2.19 ± 1.17	9.36 ± 5.52	2.14 ± 0.93	2.80 ± 1.60	20.47 ± 13.70
Fujian	0.62 ± 0.26	0.45 ± 0.24	2.67 ± 1.56	0.60 ± 0.26	0.58 ± 0.33	5.77 ± 3.99
Jiangxi	1.03 ± 0.43	1.63 ± 0.89	7.39 ± 4.36	0.99 ± 0.42	2.09 ± 1.20	16.18 ± 10.41
Shandong	2.22 ± 0.94	0.77 ± 0.44	9.59 ± 5.66	2.09 ± 0.90	0.99 ± 0.56	21.06 ± 13.54
Henan	2.28 ± 0.95	0.86 ± 0.47	8.31 ± 4.90	2.19 ± 0.96	1.10 ± 0.62	18.48 ± 12.18
Hubei	1.66 ± 0.70	1.29 ± 0.69	14.18 ± 8.35	1.57 ± 0.68	1.65 ± 0.94	31.00 ± 20.74
Hunan	1.29 ± 0.54	1.24 ± 0.68	6.87 ± 4.05	1.21 ± 0.52	1.58 ± 0.90	15.01 ± 9.98
Guangdong	0.85 ± 0.36	0.71 ± 0.40	2.53 ± 1.50	0.80 ± 0.36	0.91 ± 0.52	5.63 ± 3.78
Guangxi	0.48 ± 0.20	0.77 ± 0.42	3.35 ± 1.97	0.48 ± 0.21	0.98 ± 0.56	7.29 ± 4.99
Hainan	0.29 ± 0.11	0.30 ± 0.14	0.62 ± 0.37	0.31 ± 0.14	0.38 ± 0.21	1.36 ± 0.90
Chongqing	0.79 ± 0.34	1.79 ± 0.96	3.19 ± 1.88	0.74 ± 0.32	2.29 ± 1.30	6.98 ± 4.73
Sichuan	0.64 ± 0.26	1.01 ± 0.53	2.32 ± 1.37	0.62 ± 0.28	1.29 ± 0.74	5.05 ± 3.38
Guizhou	0.72 ± 0.30	0.99 ± 0.54	1.43 ± 0.85	0.67 ± 0.29	1.26 ± 0.72	3.10 ± 2.06
Yunnan	0.17 ± 0.08	0.31 ± 0.16	0.81 ± 0.48	0.16 ± 0.07	0.39 ± 0.22	1.79 ± 1.19
Tibet	0 ± 0	0.01 ± 0.01	0 ± 0	0 ± 0	0.01 ± 0.01	0 ± 0
Shaanxi	0.65 ± 0.28	0.59 ± 0.32	2.09 ± 1.23	0.60 ± 0.25	0.75 ± 0.43	4.61 ± 3.09
Gansu	0.24 ± 0.10	0.29 ± 0.17	0.92 ± 0.55	0.22 ± 0.09	0.38 ± 0.22	1.98 ± 1.30
Qinghai	0.08 ± 0.03	0.21 ± 0.10	0.68 ± 0.40	0.07 ± 0.03	0.26 ± 0.15	1.48 ± 0.98
Ningxia	0.66 ± 0.28	0.55 ± 0.29	1.11 ± 0.66	0.61 ± 0.26	0.71 ± 0.41	2.42 ± 1.69
Xinjiang	0.07 ± 0.03	0.10 ± 0.05	1.41 ± 0.83	0.06 ± 0.03	0.12 ± 0.07	3.08 ± 2.02

Supplementary Table 8. Major air pollution control measures implemented in the three industrial sectors from 2005 to 2019 in China.

Sector			2005	2006	2007	2008	2009	2010	2011	2012	2013	2014	2015	2016	2017	2018	2019
Power	Coal-fired power plants	Emission standard	GB 13223-2003							GB 13223-2011			ULE standard				
	Other thermal power plants	Emission standard	GB 13223-2003							GB 13223-2011							
Iron and steel	Sintering	Emission standard	GB 9078-1996							GB 28662-2012						Special emission limits in Beijing-Tianjin-Hebei and surrounding areas	Opinions on implementation of ULE
	Ironmaking	Emission standard	GB 9078-1996							GB 28663-2012						Special emission limits in Beijing-Tianjin-Hebei and surrounding areas	Opinions on implementation of ULE
	Steelmaking	Emission standard	GB 9078-1996							GB 28664-2012						Special emission limits in Beijing-Tianjin-Hebei and surrounding areas	Opinions on implementation of ULE
	Steel rolling	Emission standard	GB 9078-1996							GB 28665-2012						Special emission limits in Beijing-Tianjin-Hebei and surrounding areas	Opinions on implementation of ULE
Cement		Emission standard	GB 4915-2004									GB 4915-2013					

Supplementary Table 9. Implemented PM emission standards implemented in the power sector in the different regions, including China, European Union, and United States.

Pollutants	Types	China (2011 standard)[9]	China (ULE standard)[11]	European Union (2017 standard)				United States (MATS)
				<100 MW	100-300 MW	>300 MW, PC boiler	>300 MW, CFB	
PM (mg/m^3)	New plants	30	10	2-5				22.5
	Existing plants			2-18	2-14	2-10	2-8	

Notes: MATS, Mercury and Air Toxics Standards;

PC boiler, pulverized coal-fired boiler;

CFB boiler, circulating fluidized bed boiler.

Supplementary Table 10. Implemented PM emission standards targeting the major iron and steel production processes in the different regions, including China, European Union, and United States.

Process	Emission source	China (2012 standard)[14-17]		China (ULE standard)[19]		European Union (2012 standard)	United States (2020 standard)	
		New plants	Existing plants	New plants	Existing plants	New/Existing plants	New plants	Existing plants
Sintering	Sinter machine head	50	80	10	10	15[a] 40[b]	22.90	45.79
	Sinter machine tail	30	50	10	10	200	22.90	45.79
	Other production equipment	30	50	10	10	200	22.90	45.79
Ironmaking	Hot stove	20	50	10	10	10	6.86	22.88
	Blast ore tank	25	50	10	10	20		
	Blast furnace casting	25	50	10	10	15		
Steelmaking (basic oxygen furnaces)	Primary gas emissions	50	100			10-30[c] 50[d]		
	Hot metal pretreatment	20	50	10	10	1-10[c] 20[d]		
	Secondary gas emissions	20	50	10	10	1-10[c] 20[d]	6.87	22.90
Steelmaking	Electric arc furnace	20	50	10	10	1-10[c] 20[d]	4.58	11.45
Steel rolling	Reheating furnace	20	30	10	10			

Notes: [a] Target limit for PM emissions from sintering when equipped with bag filters as dedicated devices.

[b] Target limit for PM emissions from sintering when equipped with an advanced electrostatic precipitator.

[c] Target limit for PM emissions from steelmaking when equipped with dry dedusting technology, such as electrostatic precipitators and bag filters.

[d] Target limit for PM emissions from steelmaking when equipped with wet dedusting technology, such as wet electrostatic precipitators or scrubbers).

Supplementary Table 11. Implemented PM emission standards targeting the major clinker and cement production processes in the different regions, including China, European Union, and United States.

Emission source	China (2013 standard)[23]		European Union (BAT)	United States (NSPS)		United States (NESHAP)	
	Key region	Other region		New plants	Existing plants	New plants	Existing plants
Cement kiln	20	30	10-20	4	14	4	14
Clinker cooler	20	30	10-20	4	14	4	14
Cement grinding	10	20	10-20				
Other equipment production	10	20	10				

Notes:

BAT, best available techniques;

NSPS, new source performance standards;

NESHAP, national standards for hazardous air pollutants.

Supplementary Table 12. Model performance statistics for the comparison between the simulated and observed $PM_{2.5}$ concentrations in China in 2019.

Variable	Spring	Summer	Autumn	Winter	Average	Model performance criteria
Mean SIM (μg/m^3)	28.6	21.9	40.3	50.9	35.5	
Mean OBS (μg/m^3)	36.2	21.9	34.7	61.6	38.6	
NMB (%)	-21%	0%	16%	-17%	-8%	
NME (%)	34%	41%	36%	31%	34%	
MFB (%)	-32%	-15%	6%	-24%	-16%	± 60%
MFE (%)	43%	44%	35%	38%	40%	± 75%

Supplementary Table 13. Primary $PM_{2.5}$ emissions from iron and steel plants, power plants, and cement plants (Unit: kt).

Source	No ULE	ULE standards
Power plants	1160.9 ± 34.8	161.1 ± 4.8
Cement plants	784.7 ± 204.0	217.8 ± 56.6
Iron and steel plants	1383.5 ± 345.9	640.5 ± 160.1

Note: Regarding the iron and steel and cement industries, the scenario "No ULE" refers to the baseline year of 2019, while regarding the power plants, the scenario "ULE" refers to the baseline year of 2014.

Supplementary Table 14. Parameters of the PM emission control technologies at coal-fired power plants.

Control technology	Removal rate (%)	Capital cost (CNY/kW)	FOM cost (CNY/kWh)	Average unit cost (CNY/t)
FF	99	40–80	0.002–0.005	30–80
Dry-ESP	93	50–100	0.0004–0.002	
Wet-ESP	95	30–70	0.0003–0.002	
FF + ESP	99.9	45–90	0.001–0.004	

Notes: FF, Fabric filter;

Dry-ESP, dry method of electrostatic precipitators;

Wet-ESP, wet method of electrostatic precipitators;

FF+ESP, combination of FF and ESP technologies.

Supplementary Table 15. Parameters of the PM emission control technologies in the iron and steel industry.

Process	Technology	Capital cost (million CNY)	FOM cost (million CNY/year)	Reduction emission (t/year)	Unit cost (CNY/t)	Reference
Steelmaking	ESP	72.71	26.03	32000	1054	Dissertation of Government Document in 2012[a]
Steel rolling	HEFF	22.43	7.20	1000	9579	

Notes: HEFF, high-efficiency fabric filter;

[a] Government document, namely, Advanced Applicable Technical Guide for Energy Saving and Emission Reduction in the Steel Industry, as described on the following website: http://miit.gov.cn/n1146290/n4388791/c4237201/content.html.

Supplementary Table 16. Parameters of the PM emission control technologies in the cement industry.

Process	Technology	Removal rate (%)	Total cost (CNY/t cement clinker)
Before rotary kiln	ESP	99.50–99.97	3.97
	ESP+FF	99.80–99.99	4.24
	HEFF	99.80–99.99	4.27
After rotary kiln	ESP	99.5–99.97	5.51
	ESP+FF	99.8–99.99	4.93
	HEFF	99.80–99.99	6.66
Coal mill	HEFF	99.80–99.99	2.09
Cement mill	FF	99.00–99.50	3.53

Notes: HEFF, high-efficiency fabric filter;

ESP, electrostatic precipitator;

FF, fabric filter;

ESP+FF, combination of FF and ESP technologies.

Supplementary Table 17. Cost dataset for ULE retrofitting in the iron and steel industry.

Technology	Pollutant	Cost			
		Initial investment (10^4 CNY)	*CC* (CNY/t steel)	*FOM* (CNY/t steel)	Unit cost (CNY/t steel)
Activated carbon (coke) desulfurization and denitrification	SO_2、NO_x	32000	64	16~17	82.56
CFB + SCR	SO_2、NO_x	19000	38	15~16	55.56
Activated carbon	SO_2、NO_x	-	56.16~133.17	-	94.69
Dry desulfurization and COA denitrification	SO_2, NO_x, PM	-	30.09	-	30.09
FF	PM	-	2.56~5.68	-	4.12
ESP	PM	-	25	-	25
Uses purified gas as fuel	SO_2, NO_x, PM	-	-	0.6~1.35	0.98
Yard closure + dust removal	PM	-	11.73	-	11.73

Notes: CFB, circulating fluidized bed;

SCR, selective catalytic reduction;

COA, circulating oxidation absorption;

ESP, electrostatic precipitator;

FF, fabric filter.

Supplementary Table 18: Summary of in-stack international standard method for determination of PM emissions from stationary sources.

Standard method	Extraction method	Applicability
GB 5468-91	Isokinetic sampling	Measurement of smoke and dust emission from boilers
GB/T 16157-1996	Isokinetic sampling	Determination of particulates and sampling methods of gaseous pollutants from exhaust gas of stationary source
HJ/T397-2007	Isokinetic sampling	Technical specifications for emission monitoring of stationary source
ISO 23210:2009	Two-stage impactor	Determination of PM_{10} and $PM_{2.5}$ mass concentrations at stationary emission sources.
EPA Method 5	Isokinetic sampling	Determination of PM emissions from stationary sources
EPA Method 17	Isokinetic sampling	Determination of PM emissions from stationary sources
EPA Method 201A	Cyclone samplers	Determination of PM, PM_{10}, $PM_{2.5}$ emissions from stationary sources (constant sampling rate procedures)
EPA Method 202	Dry impinger	Determination of condensable PM emissions from stationary sources

Supplementary References

1. Huang, Y., et al. Quantification of Global Primary Emissions of PM2.5, PM10, and TSP from Combustion and Industrial Process Sources. *Environ. Sci. Technol.* 48, 13834-13843 (2014).

2. Zhu, Y., et al. Sources of particulate matter in China: Insights from source apportionment studies published in 1987–2017. *Environ. Int.* 115, 343-357 (2018).

3. Zheng, H., et al. Transition in source contributions of PM2.5 exposure and associated premature mortality in China during 2005–2015. *Environ. Int.* 132, 105111-105124 (2019).

4. Emission standard of air pollutants for thermal power plants GB 13223-1991 (in Chinese). In China Environmental Press: 1991.

5. Emission standard of air pollutants for thermal power plants GB 13223-1996 (in Chinese). In China Environmental Press: 1996.

6. Emission standard of air pollutants for thermal power plants GB 13223-2003 (in Chinese). In China Environmental Press: 2003.

7. Emission standard of air pollutants for thermal power plants GB 13223-2011 (in Chinese). In China Environmental Press: 2011.

8. Zheng, B., et al. Trends in China's anthropogenic emissions since 2010 as the consequence of clean air actions. *Atmos. Chem. Phys.* 18, 14095-14111 (2018).

9. Zhang, Q., et al. Drivers of improved PM2.5 air quality in China from 2013 to 2017. *Proc. Natl. Acad. Sci. U.S.A* 116, 24463-24469 (2019).

10. Liu, X., et al. Updated Hourly Emissions Factors for Chinese Power Plants Showing the Impact of Widespread Ultralow Emissions Technology Deployment. *Environ. Sci. Technol.* 53, 2570-2578 (2019).

11. Tang, L., et al. Substantial emission reductions from Chinese power plants after the introduction of ultra-low emissions standards. *Nat. Energy* 4, 929-938 (2019).

12. Discharge standard of pollutants for iron and steel industry GB 4911-1985 (in Chinese). In China Environmental Press: 1985.

13. Emission standard of air pollutants for industrial kiln and furnace GB 9078-1996 (in Chinese). In China Environmental Press: 1996.

14. Emission Standard of Air Pollutants for Sintering and Pelletizing of Iron and Steel Industry GB 28662-2012 (in Chinese). In China Environmental Press: 2012.

15. Emission Standard of Air Pollutants for Iron Smelt Industry GB 28663-2012 (in Chinese). In China Environmental Press 2012.

16. Emission Standard of Air Pollutants for Steel Smelt Industry GB 28664-2012 (in Chinese). In China Environmental Press: 2012.

17. Emission Standard of Air Pollutants for Steel Rolling Industry GB 28665-2012 (in Chinese). In China Environmental Press: 2012.

18. Bo, X., et al. Effect of strengthened standards on Chinese ironmaking and steelmaking emissions. *Nat. Sustain.* 4, 811-820 (2021).

19. Ministry of Ecology and Environment of the People's Republic of China. Opinions on Promoting the Implementation of Ultra-low Emissions in the Iron and Steel Industry (In Chinese). In 2019.

20. Emission standard of air pollutants for cement industry GB 4915-1985 (in Chinese). In China Environmental Press: 1985.

21. Emission standard of air pollutants for cement industry GB 4915-1996 (in Chinese). In China Environmental Press: 1996.

22. Emission standard of air pollutants for cement industry GB 4915-2004 (in Chinese). In China Environmental Press: 2004.

23. Emission Standard of Air Pollutants for Cement Industry GB 4915-2013 (in Chinese). In China Environmental Press: 2013.

24. Liu, J., et al. Carbon and air pollutant emissions from China's cement industry 1990–2015: trends, evolution of technologies, and drivers. *Atmos. Chem. Phys.* 21, 1627-1647 (2021).

25. Tang, L., et al. Plant-level real-time monitoring data reveal substantial abatement potential of air pollution and CO2 in China’s cement sector. *One Earth* 5, 892-906 (2022).

26. Li, Z.; Hanaoka, T. Plant-level mitigation strategies could enable carbon neutrality by 2060 and reduce non-CO2 emissions in China’s iron and steel sector. *One Earth* 5, 932-943 (2022).

27. *Steel Statistical Yearbook 2020*. World Steel Association: 2021.

28. Wang, X., et al. A unit-based emission inventory of SO2, NOx and PM for the Chinese iron and steel industry from 2010 to 2015. *Sci. Total Environ.* 676, 18-30 (2019).

29. Association, C. I. S., *China Steel Yearbook 2020 (In Chinese)*. 2020.

30. Guo, Z., et al. Heterogeneous Variations on Historical and Future Trends of CO2 and Multiple Air Pollutants from the Cement Production Process in China: Emission Inventory, Spatial–Temporal Characteristics, and Scenario Projections. *Environ. Sci. Technol.* 56, 14306-14314 (2022).

31. Guo, Z., et al. Field measurements on emission characteristics, chemical profiles, and emission factors of size-segregated PM from cement plants in China. *Sci. Total Environ.*, 151822-151831 (2021).

32. International Organization for Standardization (ISO). ISO 23210: 2009. Stationary source emissions — Determination of PM10/PM2,5 mass concentration in flue gas — Measurement at low concentrations by use of impactors. In International Organization for Standardization: Geneva, Switzerland: 2009.

33. United States Environmental Protection Agency (US EPA). Method 5- Determination of PM emissions from stationary sources. In US EPA: Washington, D.C.: 2020.

34. United States Environmental Protection Agency (US EPA). Method 17-Determination of PM emissions from stationary sources. In US EPA: Washington, D.C.: 2017.

35. United States Environmental Protection Agency (US EPA). Method 201A-Determination of PM10 and PM2.5 emissions from stationary sources. In US EPA: Washington, D.C.: 2010.

36. United States Environmental Protection Agency (US EPA). Method 202-Dry impinger method for determining condensable particulate emissions from stationary sources. In US EPA: Washington, D.C.: 2010.

37. Wu, B., et al. Effects of Wet Flue Gas Desulfurization and Wet Electrostatic Precipitators on Emission Characteristics of Particulate Matter and Its Ionic Compositions from Four 300 MW Level Ultralow Coal-Fired Power Plants. *Environ. Sci. Technol.* 52, 14015-14026 (2018).

38. Liu, W., et al. Migration and Emission Characteristics of Ammonia/Ammonium through Flue Gas Cleaning Devices in Coal-Fired Power Plants of China. *Environ. Sci. Technol.* 54, 390-399 (2020).

39. Tsai, J.-H., et al. Chemical constituents in particulate emissions from an integrated iron and steel facility. *J Hazard Mater* 147, 111-119 (2007).

40. Guo, Y., et al. Chemical profiles of PM emitted from the iron and steel industry in northern China. *Atmos. Environ.* 150, 187-197 (2017).

41. Guo, Z., et al. Field measurements on emission characteristics, chemical profiles, and emission factors of size-segregated PM from cement plants in China. *Sci. Total Environ.*, 151822 (2021).

42. Ding, X., et al. Unexpectedly Increased Particle Emissions from the Steel Industry Determined by Wet/Semidry/Dry Flue Gas Desulfurization Technologies. *Environ. Sci. Technol.* 53, 10361-10370 (2019).

43. Ding, X., et al. Gaseous and Particulate Chlorine Emissions from Typical Iron and Steel Industry in China. *J. Geophys. Res-Atmos.* 125, e2020JD032729 (2020).

44. Zhang, J., et al. Emission characteristics of heavy metals from a typical copper smelting plant. *J. Hazard. Mater.* 424, 127311-127319 (2022).

45. Ding, X., et al. Direct Observation of Sulfate Explosive Growth in Wet Plumes Emitted From Typical Coal-Fired Stationary Sources. *Geophys Res Lett* 48, e2020GL092071 (2021).

46. Zhang, Y., et al. Characteristics of Particulate Carbon Emissions from Real-World Chinese Coal Combustion. *Environ. Sci. Technol.* 42, 5068-5073 (2008).

47. Chow, J. C., et al. Quality assurance and quality control for thermal/optical analysis of aerosol samples for organic and elemental carbon. *Anal. Bioanal. Chem.* 401, 3141-3152 (2011).

48. Chow, J. C., et al. The IMPROVE_A Temperature Protocol for Thermal/Optical Carbon Analysis: Maintaining Consistency with a Long-Term Database. *J. Air Waste Manage. Assoc.* 57, 1014-1023 (2007).

49. Wu, D., et al. Primary Particulate Matter Emitted from Heavy Fuel and Diesel Oil Combustion in a Typical Container Ship: Characteristics and Toxicity. *Environ. Sci. Technol.* 52, 12943-12951 (2018).

50. Jiang, Y., et al. Seasonal atmospheric deposition and air–sea gas exchange of polycyclic aromatic hydrocarbons over the Yangtze River Estuary, East China Sea: Implications for source–sink processes. *Atmos. Environ.* 178, 31-40 (2018).

51. Wu, Z., et al. Air–sea exchange and gas–particle partitioning of polycyclic aromatic hydrocarbons over the northwestern Pacific Ocean: Role of East Asian continental outflow. *Environ. Pollut.* 230, 444-452 (2017).

52. Jin, L., et al. Contributions of City-Specific Fine Particulate Matter (PM2.5) to Differential In Vitro Oxidative Stress and Toxicity Implications between Beijing and Guangzhou of China. *Environ. Sci. Technol.* 53, 2881-2891 (2019).

53. Appel, K. W., et al. The Community Multiscale Air Quality (CMAQ) model versions 5.3 and 5.3.1: system updates and evaluation. *Geosci. Model Dev.* 14, 2867-2897 (2021).

54. Zheng, H., et al. Development of a unit-based industrial emission inventory in the Beijing–Tianjin–Hebei region and resulting improvement in air quality modeling. *Atmos. Chem. Phys.* 19, 3447-3462 (2019).

55. Chang, X., et al. Full-volatility emission framework corrects missing and underestimated secondary organic aerosol sources. *One Earth* 5, 403-412 (2022).

56. Zhao, B., et al. Change in household fuels dominates the decrease in PM2.5 exposure and premature mortality in China in 2005–2015. *Proc. Natl. Acad. Sci. U.S.A.* 115, 12401-12406 (2018).

57. Dong, Z., et al. Regional transport in Beijing-Tianjin-Hebei region and its changes during 2014–2017: The impacts of meteorology and emission reduction. *Sci. Total Environ.* 737, 139792-139801 (2020).

58. EPA, U. Guidance on the Use of Models and Other Analyses for Demonstrating Attainment of Air Quality Goals for Ozone, PM2.5, and Regional Haze. (2007).

59. Zhang, F., et al. Estimation of abatement potentials and costs of air pollution emissions in China. *J. Environ. Manage.* 260, 110069-110080 (2020).

60. Ministry of Ecology and Environment of the People's Republic of China *Technical Specification for Ultra-low Emission Flue Gas Treatment Works for Coal-fired Power Plants (Draft for Comments)*; Ministry of Ecology and Environment of the People's Republic of China,: Beijing, 2017.

61. Department of ecology and environment of Hebei province *Ultra-low emission standard of air pollutants for iron and steel industry (draft for comment) preparation notes*; Department of ecology and environment of Hebei province,: Shijiazhuang, 2019.

62. Li, S., et al. Emission trends of air pollutants and CO2 in China from 2005 to 2021. *Earth Syst. Sci. Data* 15, 2279-2294 (2023).

63. The NCAR Command Language (Version 6.4.0) [Software]. (2017).

Boulder, Colorado: UCAR/NCAR/CISL/VETS. http://dx.doi.org/10.5065/D6WD3XH5.
